# An integrated structural and functional profiling approach uncovers the mechanisms of pregnane X receptor binding and activation by pharmaceutical and environmental compounds

**DOI:** 10.1016/j.molpha.2026.100140

**Published:** 2026-06-24

**Authors:** Quentin Derosa, Coralie Carivenc, Savannah Sirounian, Marina Grimaldi, Abdelhay Boulahtouf, Vanessa Delfosse, Patrick Balaguer, William Bourguet

**Affiliations:** 1 Institut de Recherche en Cancérologie de Montpellier, Inserm U1194, Montpellier, France; 2 Center for Structural Biology (CBS), University of Montpellier, Inserm, CNRS, Montpellier, France

**Keywords:** Pregnane X receptor, Xenobiotics, Reporter cell lines, Proliferation, Drug-drug interactions, X-ray crystallography

## Abstract

The pregnane X receptor (PXR) is a key chemosensory protein that helps the organism adapt to its chemical environment. Indeed, humans are continuously exposed to a wide range of external chemicals, known as xenobiotics, which include environmental pollutants, food components, cosmetics, and pharmaceuticals. PXR has the unique property to sense a large variety of xenobiotics and regulate the expression of detoxifying enzymes and transporters, facilitating the clearance of these chemicals. However, prolonged activation of this pathway can lead to negative effects, such as drug-drug interactions, chemoresistance, endocrine disruption, a heightened risk of metabolic diseases, or enhanced cell growth and tumor aggressiveness. Understanding how PXR interacts with xenobiotics is crucial for predicting, assessing, and preventing the potential impacts of these chemicals on human health. Here, we present the structural and functional analysis of the interaction of PXR with 2 approved drugs (nimodipine and liranaftate), a natural flavor commonly used in cosmetics and the food industry (sclareol), and an environmentally relevant halogenated derivative of the emblematic endocrine disruptor bisphenol A (2,2'-dichlorobisphenol A). Cell-based assays show that these 4 compounds display different PXR binding potencies, stimulate the expression of key target genes related to drug metabolism and cell proliferation, and promote colon cancer cell proliferation to varying degrees. Crystallographic analysis reveals their distinct mechanisms of binding to PXR. The integrated functional and structural characterization pipeline we have established yields critical insights for both environmental risk assessment and the rational development of industrial and pharmaceutical compounds with minimal harmful PXR activity.

**Significance Statement:**

The pregnane X receptor is an off-target of many pharmaceuticals and industrial chemicals whose activation is associated with clinically relevant drug-drug interactions. This study describes a pipeline for the identification and characterization of pregnane X receptor ligands, designed to support environmental risk assessment and the informed design of safer substitutes.

## 1 Introduction

The pregnane X receptor (PXR or NR1I2 according to its official nomenclature) is a ligand-dependent transcription factor that belongs to the nuclear receptor superfamily and plays a role in various cellular mechanisms.^1^^,^^2^ Primarily expressed in the liver and intestine, 2 organs essential for xenobiotic elimination, PXR is recognized as one of the key chemosensory proteins in the body.^3^ PXR forms heterodimers with retinoid X receptors (RXR*α*, *β*, *γ*, or NR2B1-3 as per official nomenclature) and binds to PXR responsive elements in the regulatory regions of target genes. Upon activation by xenobiotics (eg, environmental contaminants, drugs, dietary components), PXR interacts with coactivators, such as the steroid receptor coactivator-1, and transcriptionally upregulates the expression of a wide array of genes, including those involved in the 3 phases of detoxification, such as *CYP3A4*, *CYP24A1*, and *CYP24A1* (metabolizing enzymes from the cytochrome P450 family), *UGT1A1* (a conjugation enzyme), and the *MDR1* transporter, among others.^4^, ^5^, ^6^ Hence, one of the primary functions of PXR is to protect the body from external chemical threats.^7^ However, sustained activation of PXR is known to lead to negative effects, including drug-drug interactions, resistance to cancer therapy,^8^^,^^9^ disruptions in the regulation of endogenous hormones,^10^ increased cell proliferation and tumor aggressiveness,^11^ as well as a higher risk of cardiovascular^12^ and metabolic diseases.^13^

Unlike classical nuclear receptors that possess high affinity–specific physiological ligands (eg, estrogen and retinoic acid receptors), PXR is activated by a variety of endogenous and exogenous compounds that differ in size and chemical structures.^14^^,^^15^ Previous crystallographic studies have revealed several unique characteristics of PXR that account for its promiscuous ligand-binding properties. These specific features include a very large and conformable ligand-binding pocket (LBP),^16^^,^^17^ a unique phenylalanine-tryptophan-tyrosine triad (F288/W299/Y306) forming a *π*-trap deeply buried within the LBP and catching chemicals through their aromatic and hydrophobic groups, and 2 cysteine residues available for covalent coupling with chemically reactive moieties that are present in many xenobiotics such as pesticides.^18^ In addition, structural studies have revealed that the large PXR balloon-shaped LBP can be divided into 4 to 5 subpockets, with the specific use of one or more pockets depending on the size and chemical composition of the ligands.^18^^,^^19^ Finally, through extensive functional and structural analyses, we previously demonstrated that 2 small compounds can simultaneously bind to 2 distinct subpockets in a cooperative manner, and that this cobinding can lead to a synergistic activation of the receptor.^18^^,^^20^

A thorough identification and characterization of PXR external ligands is essential to prevent exposure to these chemicals, avoid excessive or inappropriate receptor activation, and mitigate their potential adverse effects on human health. Despite numerous structural studies uncovering the molecular determinants of the ligand-binding promiscuity of PXR, reliable compound-specific binding modes and affinities remain hardly predictable through computational methods. To robustly address these questions, we have built a characterization pipeline that integrates multiple experimental methodologies, including high-throughput screening, various cell-based assays, and structural analyses based on X-ray crystallography. In the present study, we used this approach to analyze the PXR binding mechanisms of 4 compounds of therapeutic or environmental relevance, and examine how these bindings affect PXR activity in: (1) reporter gene assays conducted in cervical (HeLa), colon (LS174T), and liver (HepG2) cancer cells; (2) the expression of 2 PXR target genes involved in xenobiotic metabolism (*CYP3A4*) and cell proliferation (*FGF19*); and (3) colon cancer cell proliferation.

## 2 Materials and methods

### 2.1 Chemicals

SR12813 and SPA70 were purchased from Tocris Bioscience. Nimodipine and liranaftate were obtained from Sigma-Aldrich. Nimodipine enantiomers were obtained from MedChemExpress. Bisphenol A (BPA), bisphenol AF, bisphenol C2, bisphenol E, bisphenol Z, bisphenol B, bisphenol F, bisphenol C, bisphenol PH, bisphenol S, tetrabromobisphenol A (TBrBPA), and tetrachlorobisphenol A (TClBPA), as listed in Supplemental Table 1, were obtained from Sigma-Aldrich. Halogenated bisphenols also present in this screen were kindly provided by Dr Pascal Carato (University of Poitiers, France). Sclareol, benzophenones (benzophenone, 2,4-dihydroxybenzophenone, 2,2′,4,4′-tetrahydroxybenzophenone, 2-hydroxy-4-methoxybenzophenone, 2,4,4′-trihydroxybenzophenone), and parabens (4-hydroxybenzoic acid, methyl 4-hydroxybenzoate, ethyl 4-hydroxybenzoate, propyl 4-hydroxybenzoate, butyl 4-hydroxybenzoate), as listed in Supplemental Table 2, were obtained from Sigma-Aldrich. The Prestwick Pharmaceutical library (see Supplemental Table 3 for the complete list of compounds) and the dihydropyridine derivatives used in this study were provided by Prestwick Chemical Libraries (https://www.prestwickchemical.com/). JM47 was a gift from Dr Alain Chavanieu (University of Montpellier, France). All compound stock solutions were prepared at 10 mM in DMSO.

### 2.2 Cell lines

The HG5LN-GAL4-PXR reporter cell line was established by stable transfection of pSG5-GAL4DBD (residues 1–147)-human PXR ligand-binding domain (LBD) (residues 107–434)-puromycin and GAL4-RE5-*β*Globin-luciferase-neomycin plasmids. LS174T-PXR cells were obtained through stable cotransfection of LS174T colon cancer cells with the pcDNA3.1-human PXR (1–434)-neomycin plasmid and the XREM-CYP3A4-luciferase-hygromycin reporter plasmid. HepG2-PXR cells were obtained by stable cotransfection of pcDNA3-human PXR-neomycin and CYP3A4-Luciferase-hygromycin plasmids. They were grown in Dulbecco’s modified Eagle medium (Invitrogen) supplemented with 5% fetal calf serum, L-glutamine, and antibiotics (Invitrogen). The maps of the plasmids are provided in the Supplemental Material.

### 2.3 Transactivation assays

HG5LN-GAL4-PXR, LS174T-PXR, and HepG2-PXR reporter cell lines were seeded in 96-well, white, opaque, flat-bottom plates (Greiner CellStar) at 80,000 cells per well in 150 *μ*L of DMEM-F12 without phenol red, supplemented with penicillin/streptomycin (1%) and dextran-coated charcoal-treated fetal calf serum (2.5%) (test medium). Compounds were added 24 hours later using an automated workstation (Biomek 3000, Beckman Coulter), and cells were incubated at 37 °C for 30 hours. Then, the medium was removed, and 50 *μ*L of test medium containing luciferin at 0.3 mM was added per well. Luciferase activity was measured for 2 seconds in intact living cells after 10-minute stabilization using a MicroBetaWallac luminometer (PerkinElmer). The toxicity of the tested chemicals was assessed with the 3-(4,5-dimethyl-2-thiazolyl)-2,5-diphenyl-2H-tetrazolium bromide (MTT) assay, which measures the cellular metabolic activity and is widely used as an indicator of cell viability. After luminescence detection, the luciferin-containing medium was removed, and 100 *μ*L of MTT solution (0.5 mg/mL) was added to each well for further cell viability assessment. The MTT-containing medium was removed 4 hours after incubation, and DMSO was added to each well. After shaking, the plates were read using the VANTAstar (BMG LABTECH) in absorbance at 540 nm. Concentrations causing a 10% decrease in cell viability were considered cytotoxic and were excluded from the analysis. Each compound was tested at various concentrations in at least 3 independent experiments. In each experiment, assays were carried out in quadruplicate for every concentration. Results are presented as mean ± SD from the 3 or more biological replicates (the exact number of biological replicates is indicated in Supplemental Table 4) and were normalized as a percentage of the maximal activation elicited by the reference PXR agonist SR12813 (3 *μ*M). Transactivation dose-response curves were fitted using the sigmoid dose-response function of GraphPad Prism software (version 10.3.1). EC_50_ values were determined by nonlinear regression of the response versus log(concentration) data using a 4-parameter sigmoidal model. The Hill slope was allowed to vary, the upper plateau was constrained to 100%, and no weighting was applied. EC_50_ values were first calculated for each biological replicate (*n* ≥ 3). The geometric mean was then computed across all experiments, and 95% confidence intervals were subsequently determined.

### 2.4 High-throughput screening

Compounds from 3 libraries (Prestwick Pharmaceutical Library, 1260 compounds; in-house bisphenols library, 51 compounds; in-house cosmetics library, 11 compounds) were evaluated for their PXR activity using a high-throughput assay in HG5LN GAL4-PXR cells. Briefly, each compound was tested at 4 concentrations (0.3, 1, 3, and 10 *μ*M) in 2 independent experiments. The complete lists of compounds and screening data are provided in Supplemental Tables 1, 2, 3, and 5. Compounds exhibiting activity were categorized as weak (10 *μ*M < EC_50_ < 100 *μ*M), moderate (1 *μ*M < EC_50_ < 10 *μ*M), or strong (EC_50_ < 1 *μ*M) activators.

### 2.5 LS174T-PXR proliferation assay (P-SCREEN)

LS174T-PXR cells were seeded in 96-well white, clear, flat-bottom plates (Greiner CellStar) at 4500 cells per well in 150 *μ*L of DMEM-F12 without phenol red, supplemented with penicillin/streptomycin (1%) and dextran-coated charcoal-treated fetal calf serum (1%) (test medium). As the selective PXR antagonist SPA70^21^ reduces the basal proliferation of cells (Supplemental Fig. 1), we added it at 100 nM in the culture medium in order to allow a more sensitive detection of ligand-induced cell growth. Simultaneously, cells were treated with increasing concentrations of different compounds for 7 days without intermediate renewal of the test medium. At the end of the 7-day incubation period, luciferase activity was first measured in living cells as described above, prior to evaluating cell proliferation using the MTT assay. Under these extended incubation conditions, the MTT signal reflects the cumulative number of metabolically active cells and can, therefore, be used as an indirect measure of cell proliferation. Each compound was tested at various concentrations in at least 3 independent experiments (the exact number of biological replicates is indicated in Supplemental Table 6). In each experiment, assays were carried out in quadruplicate for every concentration. Results are presented as mean ± SD from 3 or more biological replicates and were normalized as a percentage of the maximal activation elicited by the reference PXR agonist JM47 (300 nM). Proliferation dose-response curves were fitted using the sigmoid dose-response function of GraphPad Prism software (version 10.3.1). EC_50_ values were determined by nonlinear regression of the response versus log(concentration) data using a 4-parameter sigmoidal model (Hill slope variable) when a plateau was reached. EC_50_ values were first calculated for each biological replicate (*n* ≥ 3). The geometric mean was then computed across all experiments, and 95% confidence intervals were subsequently determined. The activation of the proliferation by PXR ligands was stable for more than 3 months for the LS174T-hPXR cell line in culture, which corresponds to 1015 passages. No passage-dependent changes in the magnitude of the proliferative response were observed.

### 2.6 Reverse-transcription polymerase chain reaction (RT-qPCR)

Total RNA was isolated and purified using the NucleoSpin RNA Plus (Macherey-Nagel) kit for LS174T-PXR cells according to the manufacturer’s protocol. The concentration and purity of isolated RNA were measured using the Nanoready Touch microvolume spectrophotometer (purchase at Dutscher SAS). cDNA was prepared with the One Script RT Mix for qPCR w/gDNAOut (Ozyme). Quantitative reverse-transcription PCR (RT-qPCR) was performed using ONEGreen FAST qPCR Premix (Ozyme) and the primers for human CYP3A4, human fibroblast growth factor 19 (FGF19), and human 28S (Supplemental Table 7). The relative amount of RNA was calculated with the 2ΔΔCT method, and gene expression was normalized using the housekeeping rRNA 28S gene.

### 2.7 Structural analysis

The human PXR LBD (residues 130–434) was expressed in fusion with a fragment of the steroid receptor coactivator-1 (678–700) to enhance PXR stability and purified as previously described.^22^ The fused steroid receptor coactivator-1 peptide was removed by thrombin cleavage prior to the last chromatographic step. Crystals of the PXR LBD complexes were obtained by mixing 1 *μ*L of protein (4 mg/mL) preincubated (1 hour) with the ligand (3 molar equivalents) and 1 *μ*L of precipitant (50 to 100 mM imidazole, pH 7.0–7.4, 8%–14% [vol/vol] isopropanol) and equilibrated against a reservoir of 500 *μ*L of precipitant. Crystals appeared in 24 to 48 hours. Diffraction data were collected on the ID23-1, ID30A-3, and ID30B beamlines at the European Synchrotron Radiation Facility.^23^ Data were processed and scaled with XDS^24^^,^^25^ and SCALA.^26^ Crystals belong to space group P43212. The structures were determined and refined using Phenix^27^ and COOT.^28^ Data collection and refinement statistics are summarized in Supplemental Table 8. Figures were prepared with PyMOL (https://pymol.org/2/).

## 3 Results

The 4 compounds (Fig. 1) investigated in this study were identified through high-throughput screening of either commercial or in-house compound libraries using the HG5LN-GAL4-PXR bioluminescent reporter cell line.^20^ Nimodipine and liranaftate were identified among the most potent PXR agonists in the Prestwick Pharmaceutical Library (Supplemental Table 5). 2,2'-dichlorobisphenol A (2,2′-diClBPA) was identified as the most active PXR agonist in a bisphenols library generated within the Partnership for the Assessment of Risks from Chemicals European project (Supplemental Table 1). Finally, sclareol was identified as a PXR agonist in an in-house library of compounds found in cosmetics (Supplemental Table 2). The 4 compounds were subsequently selected for comprehensive structural and functional characterization based on their capacity to interact with and activate PXR and on their broader environmental significance. Nimodipine belongs to the family of 1,4-dihydropyridine compounds, which are calcium channel blockers commonly used to lower systemic vascular resistance and arterial pressure.^29^ Among this class of pharmaceuticals, nimodipine is 1 of 12 drugs used currently in the clinic. Administered orally, nimodipine is used for its therapeutic benefits in cerebral diseases, including Alzheimer's and meningeal hemorrhage. It is worth noting that the nimodipine used clinically as well as in our structural and functional studies is a racemic mixture containing equal amounts of *R* and *S* enantiomers (Fig. 1). Liranaftate is a squalene epoxidase inhibitor and an antifungal agent used to treat several dermatophytes, including some tineas.^30^ In a TOX 21 compounds library high-throughput screening, liranaftate was previously shown to serve as a PXR agonist.^31^ Sclareol, a diterpene alcohol isolated from the herbal and flavor plant, clary sage, is the predominant ingredient in the refined oil of *Salvia sclarea*. As a natural flavor, sclareol is widely used in cosmetics and the food industry, and some studies have associated this compound with phytotherapy properties and biological activities (eg, antibacterial, antifungal, anti-inflammatory).^32^ Sclareol was previously identified as a constitutive androstane receptor (NR1I3) antagonist.^33^ Finally, 2,2'-dichlorobisphenol A (2,2′-diClBPA) is part of the broad family of halogenated BPA derivatives, among which the most well known members, TBrBPA and TClBPA, are commonly used as flame retardants in electronic devices and household furnishings.^34^ These 2 compounds were previously shown to act as potent peroxisome proliferator–activated receptor *γ* (PPAR*γ*, NR1C3) environmental agonists.^35^ 2,2′-diClBPA can be formed primarily through 2 pathways: the degradation of TClBPA, which occurs both in the environment and within living organisms, or via the halogenation of BPA, as hypochlorite ions present in aqueous environments can chlorinate BPA molecules.^36^

**Fig. 1:**
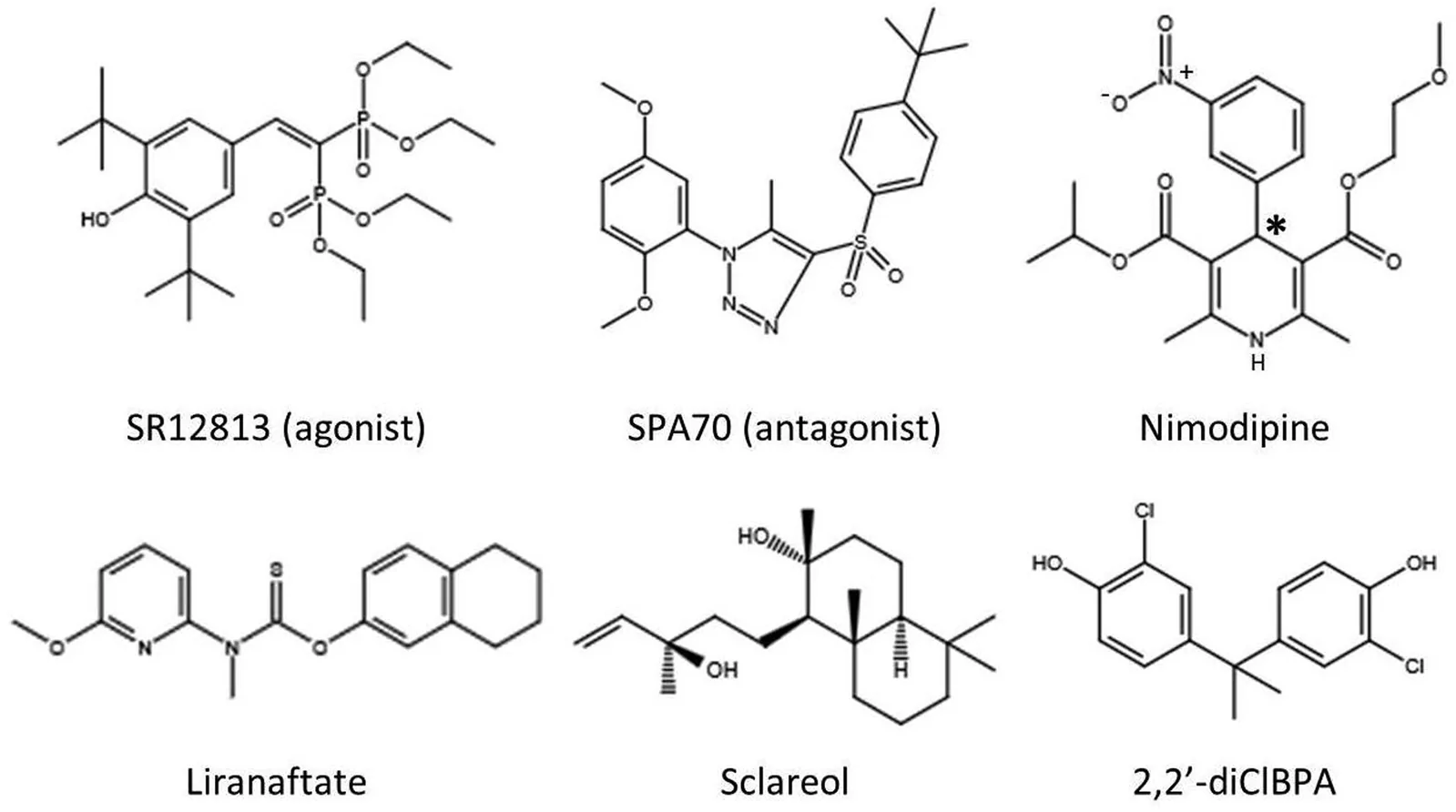
Chemical structures of compounds used in this study. (∗) depicts the asymmetric carbon atom.

### 3.1 Evaluation of the PXR transactivation properties of various compounds

We assessed the agonistic potential of the 4 chemicals in 3 different reporter cell lines. Because PXR expression can decrease the potency of compounds metabolized by PXR target gene products such as CYP3A4, UGT1A1, or MDR1, we initially used the HG5LN-GAL4-PXR reporter cell line. These cells are more sensitive to certain chemicals because the PXR chimeric receptor is unable to activate the expression of detoxifying enzymes. We next evaluated the effects of the chemicals in human colon (LS174T) and liver (HepG2) carcinoma cell lines, both of which contain full-length PXR and the CYP3A4-XREM luciferase reporter plasmids. In LS174T-PXR and HepG2-PXR cells, PXR ligands can enhance their own metabolism, leading to a reduction in their intracellular concentrations during the assay. Nevertheless, these colon and liver cell lines are more biologically relevant and better represent the cellular context in which PXR carries out its (patho)physiological functions.

The dose-response curves performed with the HG5LN-GAL4-PXR reporter cell line showed that the agonistic potentials of the compounds vary appreciably both in terms of potency and efficacy (Fig. 2A; Table 1). Nimodipine acted as a potent full agonist, activating the receptor as efficiently as the reference PXR agonist, SR12813 (EC_50_ values of 199 nM and 161 nM, respectively). In contrast, sclareol and 2,2′-diClBPA appeared as the least active compounds, with EC_50_ values in the micromolar range and a maximal activation reaching approximately 75%–80% of the transactivation level induced by SR12813. Liranaftate displayed an intermediate functional profile, acting as a full PXR agonist with an EC_50_ of 745 nM. We then characterized the activity of the compounds on colon LS174T-PXR (Fig. 2B; Table 1) and liver HepG2-PXR (Fig. 2C; Table 1) cells characterized by higher metabolic activity. Overall, nimodipine, liranaftate, and sclareol maintained similar functional profiles in the LS174T-PXR and HepG2-PXR cell lines as observed in the HG5LN-GAL4-PXR cells, suggesting minimal metabolism of these compounds during the assay. In contrast, 2,2′-diClBPA showed reduced activity in HepG2-PXR cells and an even greater activity loss in the LS174T-PXR cell line, suggesting that it undergoes substantial metabolism in these 2 cell lines. It is also noticeable that the PXR antagonist, SPA70, decreased the basal luciferase expression in all 3 cell lines (Supplemental Fig. 2; Table 1). These observations imply that the receptor, even in the absence of added ligand, may transiently assume an active conformation, or that endogenous PXR ligands are present within the cells.

**Fig. 2:**
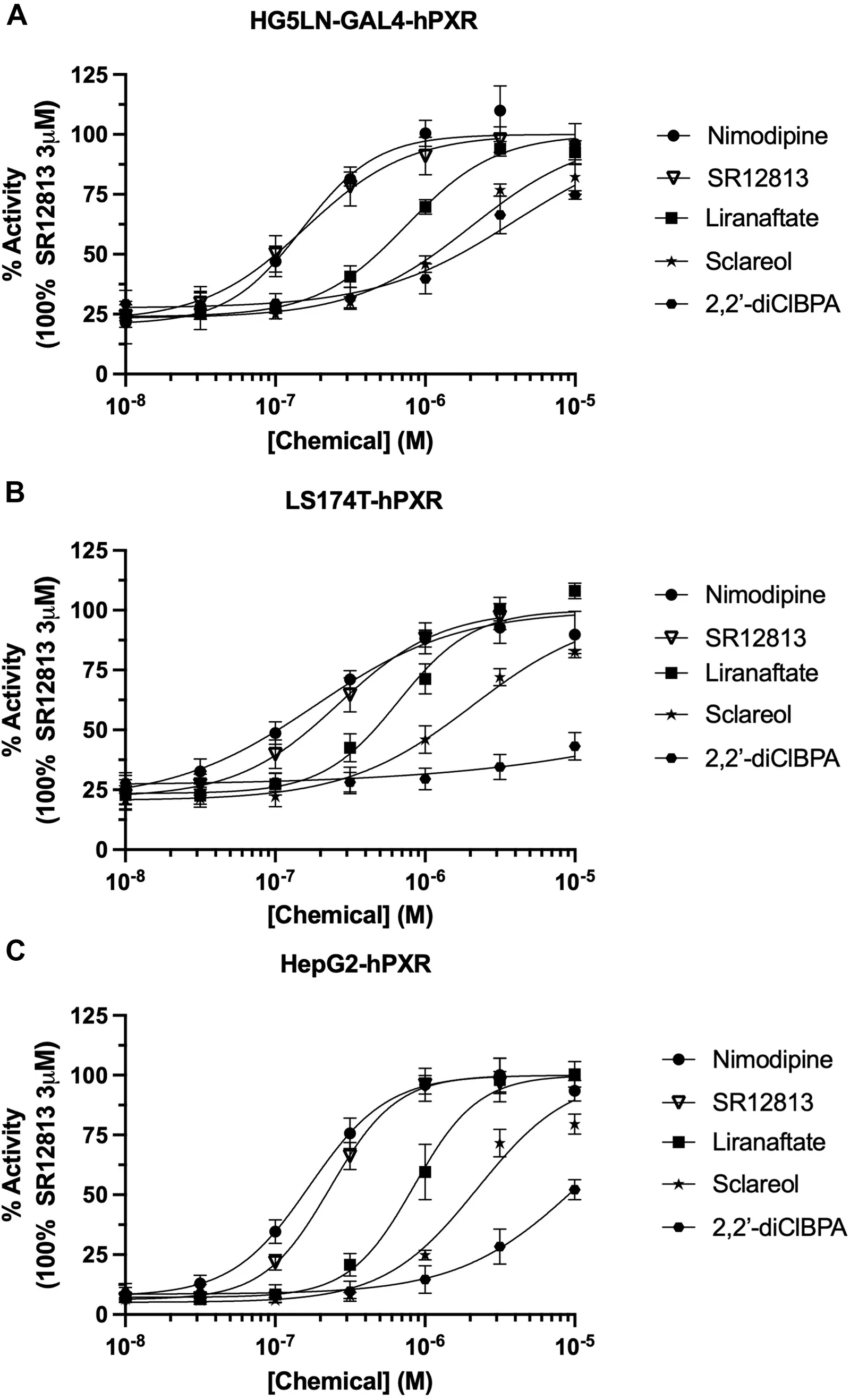
Transactivation assays. Dose-response curves of luciferase expression induced by nimodipine, SR12813, liranaftate, sclareol, and 2,2′-diClBPA in HG5LN-GAL4-PXR (A), LS174T-PXR (B), and HepG2-PXR (C) cells. Each compound was evaluated at multiple concentrations in at least 3 independent experiments conducted over 30 hours. The exact number of independent experiments performed for each compound is listed in Supplemental Table 4. For each experiment, assays were performed in quadruplicate at every concentration tested. Data are expressed as mean ± SD and normalized to the maximal activation induced by the reference PXR agonist, SR12813 (3 *μ*M). Transactivation dose-response curves were generated using the sigmoidal dose-response model implemented in GraphPad Prism software (version 10.3.1). EC_50_ values were calculated by nonlinear regression analysis of response versus log(concentration) data using a 4-parameter sigmoidal model with a variable Hill slope and the upper plateau constrained to 100%, and are reported in Table 1.

**Table 1:** EC_50_ and maximal activity values derived from the dose-response curves in Fig. 2 The IC_50_ and minimal activity values for the antagonist SPA70 are also reported. Values are the mean ± SD from at least 3 separate experiments. Basal level cell line represents the percentage of maximal SR12813 response in the absence of added ligand. For agonists, EC_50_ values were calculated by nonlinear regression analysis of response versus log(concentration) data using a 4-parameter sigmoidal model with a variable Hill slope and the upper plateau constrained to 100%. For SPA70, IC_50_ values were calculated by nonlinear regression analysis of response versus log(concentration) data using a 4-parameter sigmoidal model with a variable Hill slope, and the lower plateau was 6%, 5%, and 2% for HG5LN-GAL4-PXR, LS174T-PXR, and HepG2-PXR cells, respectively.

	HG5LN-GAL4-PXR		LS174T-PXR		HepG2-PXR	
	Basal Level Cell Line (%) 24 ± 2		Basal Level Cell Line (%) 28 ± 3		Basal Level Cell Line (%) 8 ± 1	
Agonism						
Compound	EC_50_ (nM) (95% CI)	Max (%)	EC_50_ (nM) (95% CI)	Max (%)	EC_50_ (nM) (95% CI)	Max (%)
SR12813 (Ref. agonist)	161 (142–182)	98 ± 7	268 (254–282)	99 ± 7	234 (213–256)	97 ± 5
Liranaftate	745 (582–946)	94 ± 3	659 (436–974)	108 ± 3	856 (736–985)	100 ± 5
Nimodipine	146 (96–217)	110 ± 9	183 (100–301)	93 ± 5	171 (140–207)	100 ± 5
Sclareol	2029 (1348–3083)	82 ± 7	2081 (1578–2747)	83 ± 1	2231 (1497–3438)	80 ± 4
2,2′-diClBPA	3935 (2520–6449)	75 ± 2	39500 (30780–53810)	43 ± 6	10800 (10090–11630)	52 ± 4

Antagonism						
Compound	IC_50_ (nM) (95% CI)	Min (%)	IC_50_ (nM) (95% CI)	Min (%)	IC_50_ (nM) (95% CI)	Min (%)
SPA70 (antagonist)	47 (37–60)	6 ± 1	20 (17–24)	5 ± 1	4 (1–11)	2 ± 1

Because nimodipine belongs to a large family of calcium channel blockers, we assessed the agonistic potential of several dihydropyridine derivatives currently used in the clinic. The transactivation assays demonstrated that all nimodipine analogs act as PXR agonists in the 3 reporter cell lines, with nimodipine being the most potent (Supplemental Table 9). Likewise, with several halogenated BPA analogs present in our in-house library, we could assess how halogenation alters BPA binding to and activation of PXR. Remarkably, 2,2′-diClBPA and 2,2'-BrClBPA exhibited higher activity than BPA, 2,2'diBrBPA, and tetrahalogenated BPAs (Supplemental Table 10). As for 2,2′-diClBPA, the other BPA analogs showed reduced activity in HepG2-PXR cells and almost no activity in the LS174T-PXR cell line.

### 3.2 Activity of compounds on PXR target gene expression and cancer cell proliferation

Using RT-qPCR in LS174T-PXR cells, we next evaluated the ability of each of the 4 test compounds to induce the expression of 2 well characterized endogenous target genes of PXR, namely, the cytochrome P450 *CYP3A4* and the *FGF19* involved in drug metabolism^37^ and cell proliferation,^11^ respectively. Consistent with the reporter gene assay results (Fig. 2B), all tested compounds, except 2,2′-diClBPA, increased the mRNA expression levels of both CYP3A4 and FGF19 (Fig. 3, A and B). CYP3A4 mRNA levels were induced by 20.9-, 20.3-, 29.7-, and 30.4-fold following treatment with SR12813, nimodipine, liranaftate, and sclareol, respectively. Similarly, FGF19 mRNA expression increased by 10.7-, 24.5-, 17.1-, and 19.4-fold in response to SR12813, nimodipine, liranaftate, and sclareol, respectively (Supplemental Table 11). These observations confirmed the agonistic activity of these chemicals and that 2,2′-diClBPA undergoes rapid metabolism in colon cancer cells. Given that activation of the PXR has been shown to stimulate the proliferation of human colon cancer cells, with PXR-induced *FGF19* expression playing a central role in this process,^11^ we aimed to assess the impact of various compounds on the growth of LS174T-PXR cells. To achieve this, we conducted dose-response experiments comparing the induction of cell proliferation by the reference agonist, JM47,^38^ and various test compounds using MTT assays (Fig. 4; Table 2). JM47 was selected as the reference compound because it exhibited greater proliferative efficacy than SR12813 (Supplemental Fig. 3). Consistent with the previous experiments, all compounds, except 2,2′-diClBPA, promoted cell proliferation. Nimodipine elicited the strongest effect, reaching a maximal induction of 2.4-fold, followed by liranaftate and sclareol, which produced maximal inductions of approximately 1.9- to 2.0-fold. The EC_50_ value of nimodipine in cell proliferation assays (217 nM) was comparable to that obtained in transactivation assays (183 nM) performed in the same cell line. In contrast, liranaftate and sclareol exhibited lower proliferative activity than nimodipine, in agreement with their weaker induction of luciferase expression in LS174T-PXR cells. Considering that proliferation assays span 7 days, whereas transactivation assays are limited to 30 hours, the absence of effect of 2,2′-diClBPA on proliferation suggests that this compound was fully metabolized during the extended exposure period. Complete disappearance of 2,2′-diClBPA was confirmed by measuring luciferase expression at the end of the proliferation experiment. Although all other chemicals remained active after 7 days, no luciferase expression was detected for 2,2′-diClBPA (Supplemental Fig. 4). Note in addition that, in a 7-day study, all compounds exhibited cytotoxicity at their highest administered concentrations (Fig. 4), whereas this effect was absent or lower in the 30-hour-long transactivation assays (Fig. 2, A–C).

**Fig. 3:**
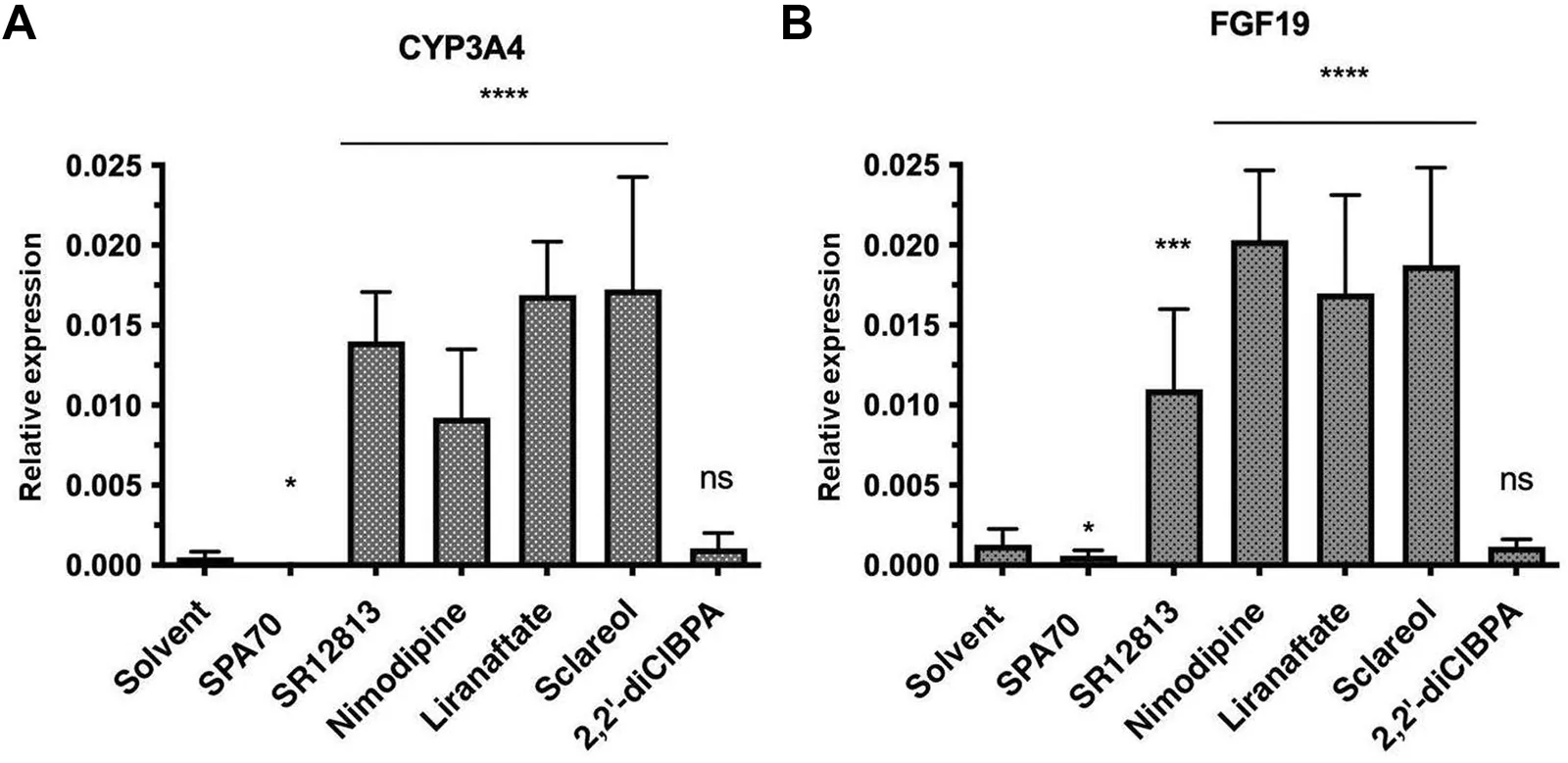
Impact of PXR ligands on PXR target gene expression in LS174T-PXR cells. RT-qPCR of CYP3A4 (A) and FGF19 (B). mRNA expression was performed in LS174T-PXR cells treated with 0.1% DMSO (negative control), SPA70 1 *μ*M, SR12813 3 *μ*M, nimodipine 10 *μ*M, liranaftate 10 *μ*M, sclareol 10 *μ*M, and 2,2′-diClBPA 10 *μ*M for 30 hours. After 30 hours, RT-qPCR for CYP3A4 and FGF19 was performed and was normalized to 28S levels. Results were obtained from 4 independent biological replicates, each measured in technical duplicates. Data are expressed as mean ± SD and compared with control cells treated with DMSO (0.1%). Statistical analysis was performed using a two-way ANOVA followed by Dunnett’s post hoc test for multiple comparisons, comparing each treatment condition to the DMSO control. *P* values indicate comparisons of indicated groups versus the untreated group (∗*P* ≤ .05, ∗∗∗*P* ≤ .001, ∗∗∗∗*P* ≤ .0001). ns, nonsignificant.

**Fig. 4:**
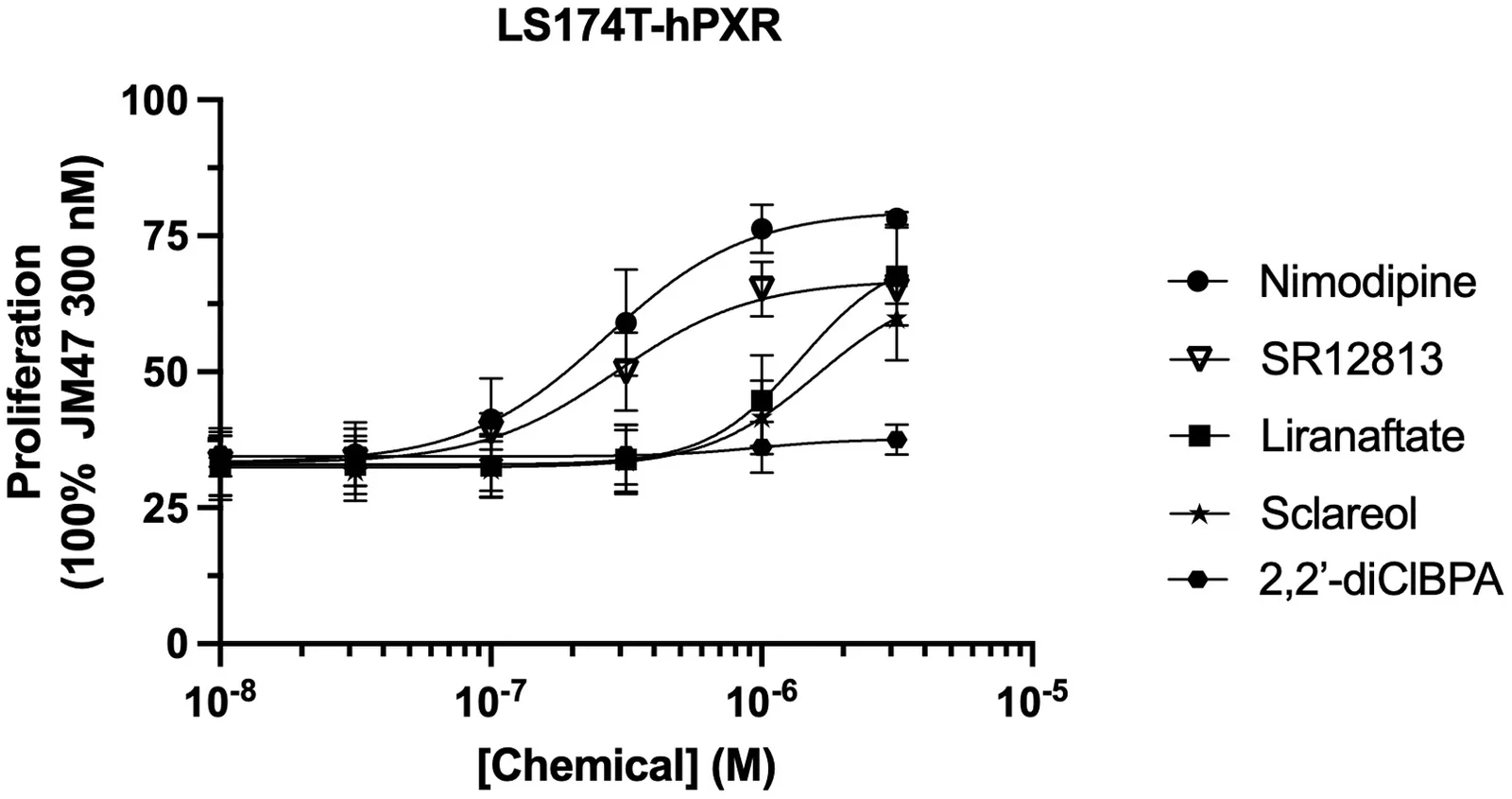
Effects of compounds on cell proliferation. Dose-response curves of proliferation induced by nimodipine, SR12813, liranaftate, sclareol, and 2,2′-diClBPA in LS174T-PXR cells. Cells were exposed to the treatments for 7 days. Each compound was tested at various concentrations in at least 3 independent experiments. Each compound was evaluated at multiple concentrations in at least 3 independent experiments conducted over 30 hours. The exact number of independent experiments performed for each compound is listed in Supplemental Table 6. Results are presented as mean ± SD, expressed as a percentage of the maximal activation elicited by the reference PXR agonist, JM47 (300 nM). Proliferation dose-response curves were fitted using the sigmoid dose-response function of GraphPad Prism software (version 10.3.1). EC_50_ values were determined by nonlinear regression of the response versus log(concentration) data using a 4-parameter sigmoidal model (Hill slope variable) for nimodipine and SR12813, where a plateau was reached (Table 2). For the other compounds (liranaftate, sclareol, and 2,2′-diClBPA), we did not calculate EC_50_ values because we did not reach a plateau. For these compounds, we only reported the maximum value reached.

**Table 2:** EC_50_ and maximal activity values derived from the dose-response curves in Fig. 4 Values are the mean ± SD from 3 separate experiments. The basal level cell line corresponds to the percentage of maximal JM47 response in the presence of 100 nM SPA70. EC_50_ values were determined by nonlinear regression of the response versus log(concentration) data using a 4-parameter sigmoidal model (Hill slope variable) for nimodipine and SR12813, where a plateau was reached. For the other compounds (liranaftate, sclareol, and 2,2′-diClBPA), we did not calculate EC_50_ values because we did not reach a plateau. For these compounds, we only reported the maximum value reached.

LS174T-PXR P-Screen Assay		
Basal Level Cell Line (%) 34 ± 2		
Compound	EC_50_ (nM) (95% CI)	Max (%)
SR12813	241 (134–328)	65 ± 5
Liranaftate	ND^a^	68 ± 9
Nimodipine	217 (159–291)	78 ± 1
Sclareol	ND^a^	60 ± 8
2,2′-diClBPA	ND^a^	38 ± 3

a Not determined due to insufficient response to allow reliable curve fitting.

### 3.3 Structural insights into the binding modes of compounds to PXR

To gain structure-based insight into the mode of action of nimodipine, liranaftate, sclareol, and 2,2′-diClBPA, we determined the crystal structures of the PXR LBD bound to each individual compound, at resolutions ranging from 2.0 to 2.9 Å (Supplemental Table 8). The structures display the canonical active conformation of nuclear receptor LBDs, with the C-terminal helix H12 (also termed AF-2 or activation helix) capping the LBP. Compounds could be placed in their respective electron density revealing different binding modes and cavity occupancies (Fig. 5, A–D). A conserved feature among the 4 structures is the involvement of the aromatic F288/W299/Y306 triad, also referred to as the *π*-trap, which captures all 4 compounds via hydrophobic or aromatic contacts (Fig. 5, E–H). Other interactions between ligands and the LBP essentially encompass van der Waals interactions with surrounding aliphatic and aromatic residues, mainly from helices H3 and H5. Previous studies have shown that the PXR LBD comprises 4 distinct subpockets. Depending on their size and chemical properties, ligands can occupy anywhere from 1 to all 4 of these subpockets.^18^ Subpockets 1 and 4 were identified as the primary binding sites for organochlorine pesticides and estrogens, respectively. In contrast, the main anchoring site for the pesticides, fipronil and oxadiazon, lies in subpocket 2, whereas pretilachlor and tributyltin predominantly occupy subpocket 3 (Fig. 5I). 2,2′-diClBPA exhibits a distinctive binding mechanism involving 2 molecules within the LBP (Fig. 5, A and E; Supplemental Fig. 5A). However, several observations indicate that these 2 molecules interact with the receptor with different strengths. A well defined electron density fully encompassing molecule #1 was observed in subpocket 1, which contains the *π*-trap. In contrast, molecule #2, located in subpocket 4 near helix H12, was associated with a weaker density. Nonetheless, the available density (especially that of chlorine atoms and methyl groups) was sufficient to position molecule #2 in the binding pocket. After refinement, the final model showed that molecule #2 had a higher mean B-factor than molecule #1 (98.6 Å^2^ vs 79.3 Å^2^), an occupancy of approximately 0.8, and an incomplete electron density (real-space correlation coefficient of 0.77). Together, these observations support a partial occupancy and increased conformational flexibility of molecule #2 within the PXR binding pocket. One phenol group of each 2,2′-diClBPA molecule and their ortho chlorine atoms are involved in hydrogen and halogen bonds with S208 or S247, respectively (Fig. 5E). Our structural studies reveal that liranaftate occupies the same binding site, and approximately the same volume, as oxadiazon (Fig. 5, B and F; Supplemental Fig. 5B), consistent with their very similar EC_50_ values of ∼ 0.7 *μ*M and ∼ 0.9 *μ*M, respectively^18^ (Table 1). The bicyclic diterpene core of sclareol occupies subpocket 1, with its hydroxyl group forming a hydrogen bond with the main-chain carbonyl of L209. Meanwhile, the aliphatic side chain projects outward from the aromatic cage toward subpocket 4, although it does not fully extend into it (Fig. 5, C and G). As previously mentioned, the nimodipine used in our structural studies is a racemic mixture (Fig. 1). We, therefore, measured the PXR binding capacity of the 2 enantiomers and found that both *R*- and *S*-nimodipine activate PXR with comparable potency (Supplemental Fig. 6, A–C; Supplemental Table 12). Accordingly, we generated a model that includes both molecules by placing the *R* and *S* enantiomers into the electron density. The *R* enantiomer was oriented with the isopropoxycarbonyl group engaging the *π*-trap, whereas the methoxyethoxycarbonyl and nitrophenyl substituents extend toward helices H11 and H12, respectively (Fig. 5, D and H). To preserve placement of the nitrophenyl ring of the *S* enantiomer within the electron density, a 180° rotation was applied to the whole molecule, resulting in localization of the methoxyethoxycarbonyl group within the aromatic cage and orientation of the isopropoxycarbonyl group toward H11 (Supplemental Fig. 7). The side chain interacting with the *π*-trap is supported by a reasonably well defined electron density, in contrast to the opposite side chain, which exhibits very weak density, reflecting substantial dynamics in this region. For clarity, the figures present the 2 enantiomers individually. However, the structure deposited in the Protein Data Bank (PDB) (PDB code 9TWE) includes both *R*- and *S*-nimodipine. With 30 non-hydrogen atoms, versus 19 (×2), 22, and 23 for 2,2′-diClBPA, sclareol, and liranaftate, respectively, nimodipine engages, at least partially, each of the 4 subpockets. This is consistent with its comparatively lower EC_50_ value among the compounds analyzed (Table 1).

**Fig. 5:**
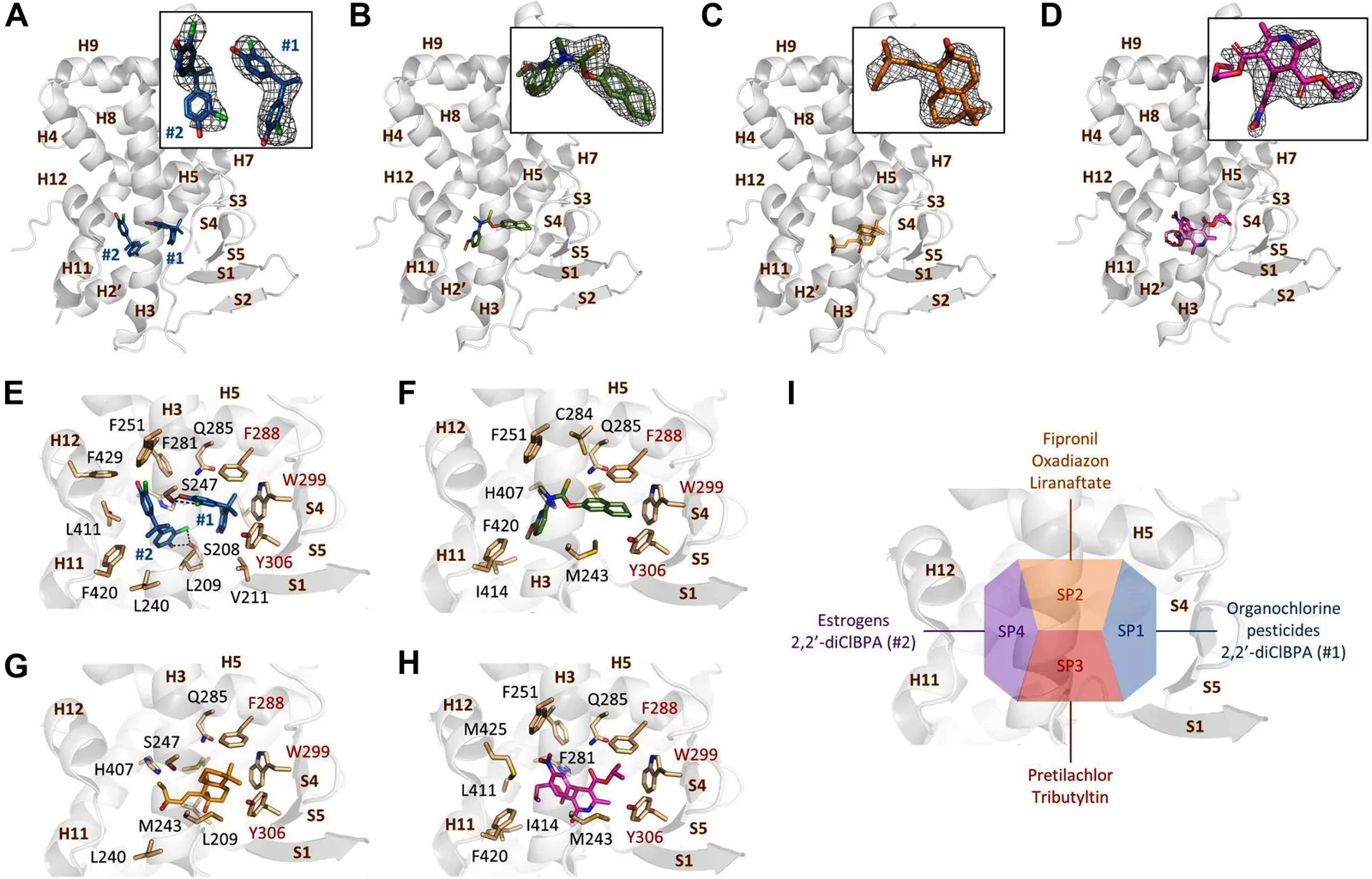
Structural basis for PXR interaction with chemicals. Overall structures of the ligand-binding domain of PXR in complex with 2,2′-diClBPA (A), liranaftate (B), sclareol (C), and nimodipine (D). The secondary structural elements of PXR LBD are labeled (*α*-helices H1–H12, *β*-strands S1–S5). The insets show the 2Fo-Fc electron density maps of each ligand. The inset of panel (D) displays *R*-nimodipine. Close-up views of the ligand-binding pocket of PXR bound to 2,2′-diClBPA (E), liranaftate (F), sclareol (G), and *R*-nimodipine (H). Key PXR residues in contact with the compounds (4.2 Å cut-off) are depicted. Amino acids of the *π*-trap are highlighted in red. (I) Shown are the 4 subpockets (SP1–SP4) constituting the PXR ligand-binding cavity, along with their associated ligands.

## 4 Discussion

Although PXR plays a major protective role against exposure to external chemical stressors, prolonged (eg, because of constant exposure to environmental pollutants) or inappropriate (eg, by a drug whose therapeutic target is not PXR) activation of this receptor can have deleterious consequences. Activation of PXR by a xenobiotic induces the expression of metabolic enzymes and transporters that promote clearance of the activating compound and can also accelerate the metabolism and elimination of other endogenous or exogenous molecules present in the body. These molecules may include endogenous hormones or therapeutic drugs, leading to reduced hormone levels and endocrine disruption, respectively, or to decreased drug exposure and the development of drug resistance. Under certain conditions and through distinct mechanisms (eg, induction of *FGF19*), PXR activation has also been implicated in metabolic disorders and cell proliferation.^2^^,^^39^ In this context, it is important to: (1) establish robust methods for identifying and characterizing the impact of exogenous molecules on PXR activity, (2) limit undesirable exposure to PXR activators, and (3) develop industrial and therapeutic molecules that lack PXR activity. In this line, many prescription drugs have been shown to exhibit substantial PXR activity, such as the anticancer agent, dabrafenib.^40^ In recent years, structural biology approaches have enabled their rational derivatization to generate safer pharmaceuticals that maintain high affinity for their intended targets while losing the capacity to bind to PXR.^41^^,^^42^

We have developed an experimental pipeline allowing for the comprehensive characterization of PXR ligands, both at the functional and structural levels. This pipeline is primarily based on the generation and use of 3 dedicated stable cell lines. The first, HG5LN-GAL4-PXR, is characterized by low metabolic activity and enables rapid screening of several hundred compounds using fully automated platforms and chemical libraries. Following this initial screening step, the activity of hit compounds is further evaluated in 2 additional cell lines, LS174T-PXR (colon carcinoma cells) and HepG2-PXR (hepatocellular carcinoma cells), which exhibit higher metabolic activity and more closely reflect the physiological contexts in which PXR functions. These cells are used to perform reporter gene assays, to measure the expression of 2 PXR target genes, *CYP3A4* and *FGF19*, implicated in key PXR functions such as xenobiotic metabolism and cell proliferation, respectively, and to assess ligand-induced cell proliferation (LS174T-PXR cells). These experiments also provide valuable information on the metabolic stability of the compounds. The functional cellular approaches are further complemented by X-ray crystallography, using a robust and recently established protocol for PXR LBD expression, purification, and crystallization,^22^ which provides atomic-level insight into the ligand-binding mechanisms through determination of 3-dimensional structures. This information, made freely accessible through deposition of structural data with the PDB, is critical for the modification and redesign of existing drugs and chemicals to reduce deleterious side effects arising from their interaction with PXR.

Here, we illustrate this approach using 4 compounds of therapeutic or environmental relevance identified through screening of chemical libraries with the HG5LN-GAL4-PXR cell line. This screen identified nimodipine, liranaftate, sclareol, and 2,2′-diClBPA as PXR agonists, with EC_50_ values ranging from ∼ 0.15 to ∼ 3.9 *μ*M (Table 1). These potencies may be of concern, as the first 3 compounds are drugs or food additives that could reach biologically relevant concentrations in the body. Notably, nimodipine displays PXR agonist activity with potency and efficacy comparable to the reference agonist, SR12813. With the exception of 2,2′-diClBPA, which appears to be rapidly metabolized, the results obtained in LS174T-PXR and HepG2-PXR cells are comparable to those observed in the HG5LN-GAL4-PXR cell line. These findings indicate a high resistance to metabolism for the other 3 compounds, as confirmed in long-term experiments in which they remained active after up to 7 days of treatment (Supplemental Fig. 4). All compounds, except 2,2′-diClBPA, were identified as strong inducers of the major drug-metabolizing enzyme, CYP3A4, and may, therefore, have the potential to cause drug-drug interactions and other metabolism-related adverse effects (Fig. 3A). We also demonstrated that these compounds induce FGF19 expression (Fig. 3B) and, consequently, promote proliferation of LS174T-PXR cancer cells (Fig. 4), potentially increasing the risk of adverse outcomes such as secondary tumor formation. Within the 1,4-dihydropyridine family of pharmaceuticals, our findings demonstrate appreciable heterogeneity in the interaction of individual compounds with PXR (Supplemental Table 9). These data suggest that isradipine and felodipine, which activate PXR with 10- to 15-fold lower potency than nimodipine, may offer a reduced risk of PXR-mediated adverse effects in clinical use.

Our structural analysis elucidated the interactions between the compounds and PXR. The 4 compounds display distinct binding modes. Notably, 2 molecules of 2,2′-diClBPA are observed within the PXR binding pocket. We have previously demonstrated that the PXR LBP can accommodate 2 ligands simultaneously and that, for certain ligand pairs, binding occurs cooperatively.^18^^,^^20^ In such cases, the binding affinity of the binary mixture is 1 to 2 orders of magnitude higher than that of individual compounds. However, we also observed that cobinding does not necessarily imply cooperative binding, as some ligand combinations, although simultaneously present in the pocket, did not exhibit increased affinity compared with the individual molecules. It is difficult to determine whether the binding of the 2 identical 2,2′-diClBPA molecules is cooperative or not. However, both the modest micromolar potency (Table 1) and weak electron density observed for molecule #2 (Fig. 5A) suggest little to no cooperativity in this case. Our cell-based assays indicate that partial halogenation enhances PXR activation by bisphenols, with a preference for dihalogenation at the 2 and 2′ positions (Supplemental Table 10). In contrast, tetrahalogenated BPA analogs such as TBrBPA and TClBPA, although exhibiting the highest potency for PPAR*γ* among halogenated BPA derivatives,^35^ display reduced activity toward PXR (Supplemental Table 10). This can be explained on structural grounds, as a conserved binding mode would place the halogen atoms at positions 6 and 6′ in steric conflict with several residues lining the binding pocket. The other 3 compounds exhibit more conventional binding mechanisms, though they differ in their utilization of the 4 subpockets, supporting their varied PXR binding capacities. The activation of PXR by several members of the dihydropyridine family of compounds, including felodipine, isradipine, and nicardipine, has been reported previously.^43^^,^^44^ As observed with nimodipine, PXR activation appeared to be nonstereospecific, with both enantiomers of each compound producing similar effects. In addition, activation of the aryl hydrocarbon receptor (AHR) by these compounds was also evaluated.^44^ Like PXR, AHR is a ligand-dependent transcription factor that can recognize a wide range of exogenous molecules, including drugs, and promote their metabolism.^45^ However, in contrast to PXR, AHR activation by dihydropyridines was found to be enantioselective.^44^ These findings likely reflect the substantial differences observed in the organization and volume of the LBPs of the 2 receptors, with that of AHR being smaller and more structurally constrained.^18^^,^^46^ Finally, these results confirm that, unlike other nuclear receptors such as steroid hormone receptors, retinoic acid receptors, or PPAR*γ*, PXR activation is not necessarily correlated with direct interactions between the ligand and the H12 activation helix.^18^ Notably, liranaftate and sclareol exhibit higher maximal activation despite lacking H12 contacts, compared with 2,2′-diClBPA, which engages in multiple van der Waals interactions with F429 (Fig. 2A; Table 1).

Taken together, the characterization pipeline described herein provides an extensive body of data that facilitates both the identification and detailed functional characterization of PXR ligands, thereby strengthening environmental risk assessment. In addition, it delivers structural insight into ligand-binding modes, informing the rational (re)design of chemical alternatives that are less prone to induce PXR-mediated adverse effects.

## Conflict of interest

The authors declare no conflicts of interest.
