# Supplementary material for: An integrated structural and functional profiling approach uncovers the mechanisms of pregnane X receptor binding and activation by pharmaceutical and environmental compounds
Source: Mol Pharmacol. 2026 Jun 24;108(8):100140. doi: 10.1016/j.molpha.2026.100140 (PMC13727653; doi:10.1016/j.molpha.2026.100140)

Supplemental material

An integrated structural and functional profiling approach uncovers the mechanisms of pregnane X receptor binding and activation by pharmaceutical and environmental compounds

Quentin Derosa[1], Coralie Carivenc[2], Savannah Sirounian[2], Marina Grimaldi[1], Abdelhay Boulahtouf[1], Vanessa Delfosse[2], Patrick Balaguer[1,*], William Bourguet[2,*]

[1]Institut de Recherche en Cancérologie de Montpellier, Inserm U1194, 34090, Montpellier, France
[2]Center for Structural Biology (CBS), Univ. Montpellier, Inserm, CNRS, Montpellier, France

[*]Corresponding authors: bourguet@cbs.cnrs.fr or patrick.balaguer@inserm.fr
Q.D. and C.C. contributed equally to this work.

This file includes:

Supplemental Figures 1 to 7

Supplemental Tables 4 and 6 to 12

Supplemental information: maps of the plasmids used to establish the PXR reporter cell lines.

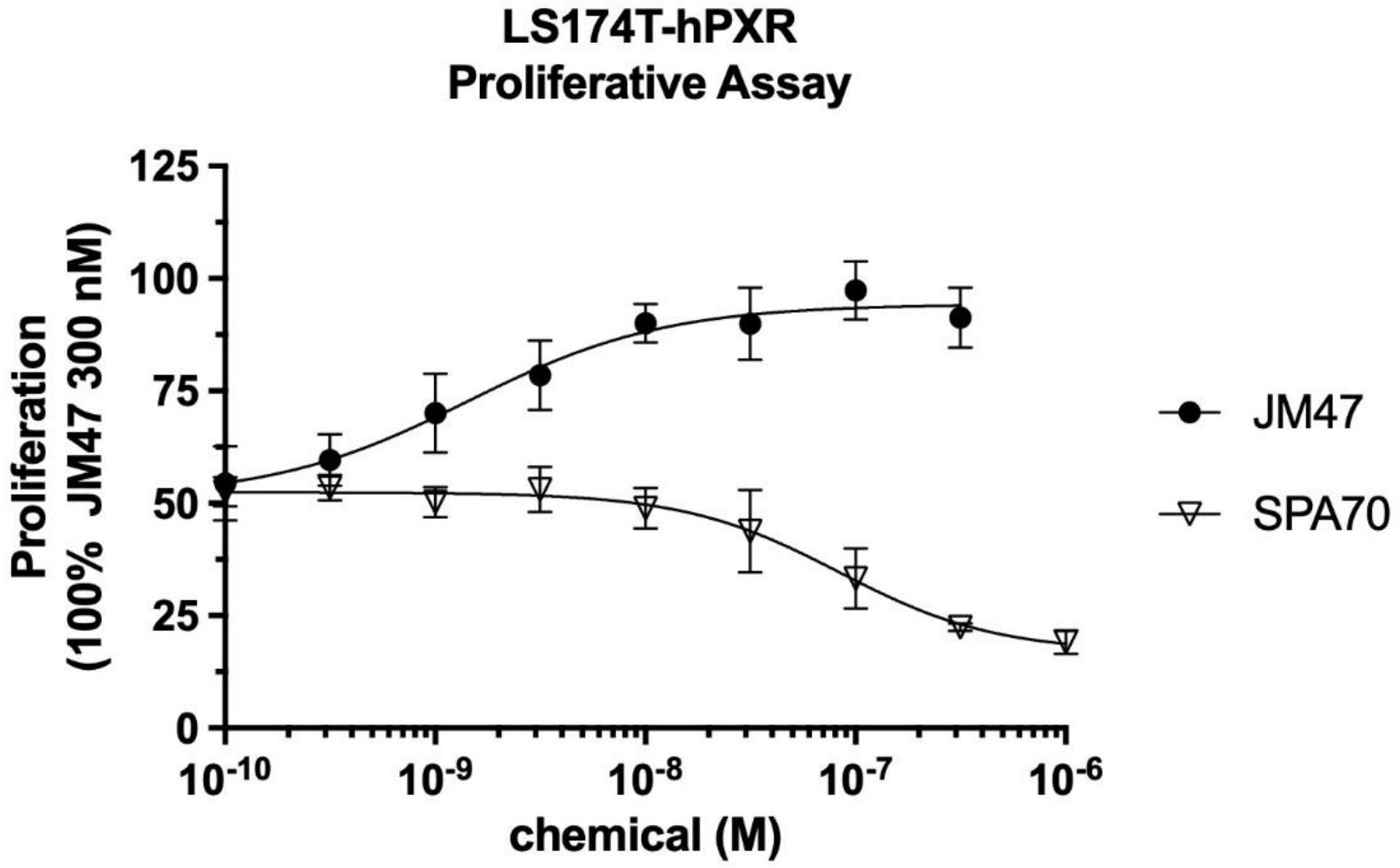

Supplemental Fig. 1. Effect of JM47 and SPA70 on proliferation of LS174T-PXR cells. Cells were exposed to the treatments for 7 days. Results are expressed as the percentage of the maximum proliferation induced by 300 nM JM47. Dose–response curves were fitted using a four-parameter sigmoidal model in GraphPad Prism. The Hill slope was treated as a variable parameter, the upper and lower asymptotes were left unconstrained, and no weighting was applied during the fitting procedure.

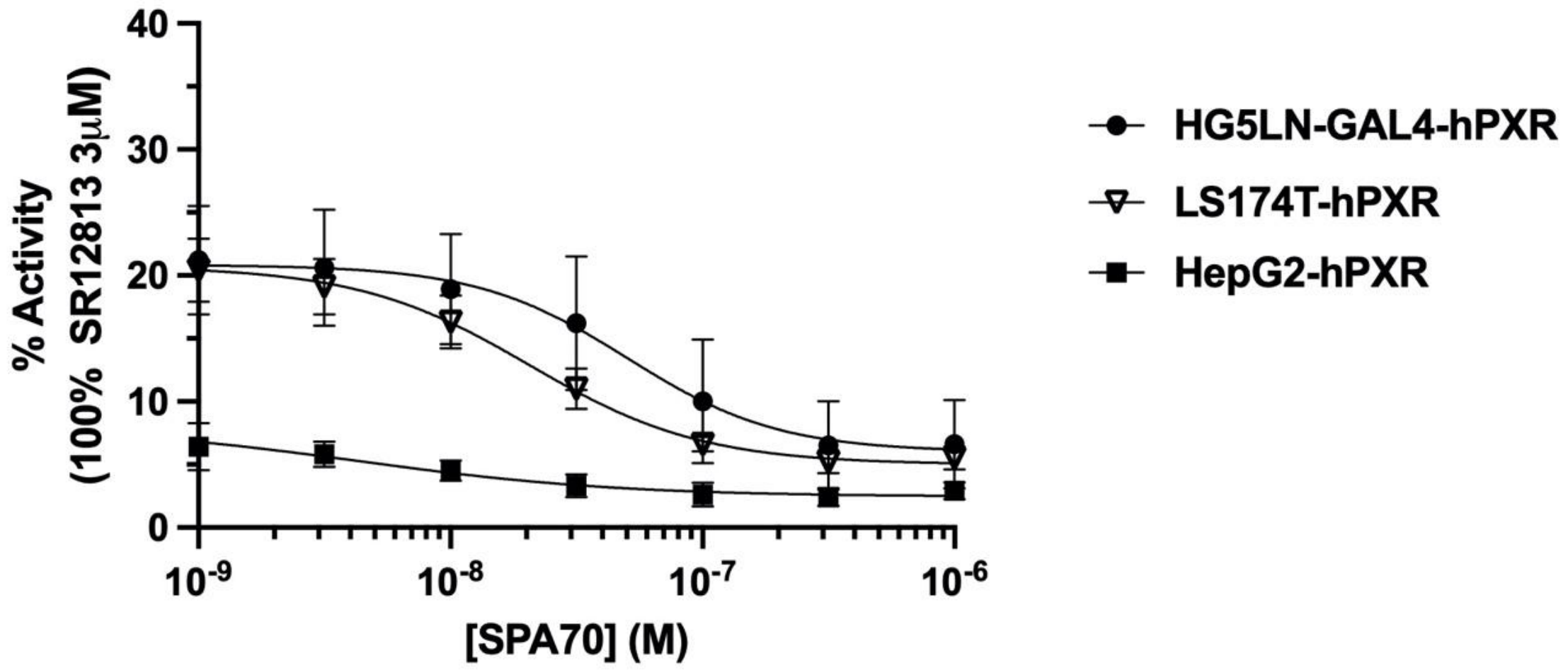

Supplemental Fig. 2. Transactivation assays. Dose-response curves of luciferase expression repressed by SPA70 in HG5LN-GAL4-PXR, LS174T-PXR and HepG2-PXR cells. SPA70 was evaluated at multiple concentrations in at least three independent experiments conducted over 30 h. For each experiment, assays were performed in quadruplicate at every concentration tested. Data are expressed as mean ± SD and normalized to the maximal activation induced by the reference PXR agonist SR12813 (3 μM). Transactivation dose–response curves were generated using the sigmoidal dose–response model implemented in GraphPad Prism. IC50 values were determined by nonlinear regression analysis of response versus log(concentration) data using a four-parameter sigmoidal model with a variable Hill slope. The lower plateau was constrained to 6%, 5%, and 2% for HG5LN-GAL4-PXR, LS174T-PXR, and HepG2-PXR cells, respectively. IC50 values and minimum activities are reported in Table 1.

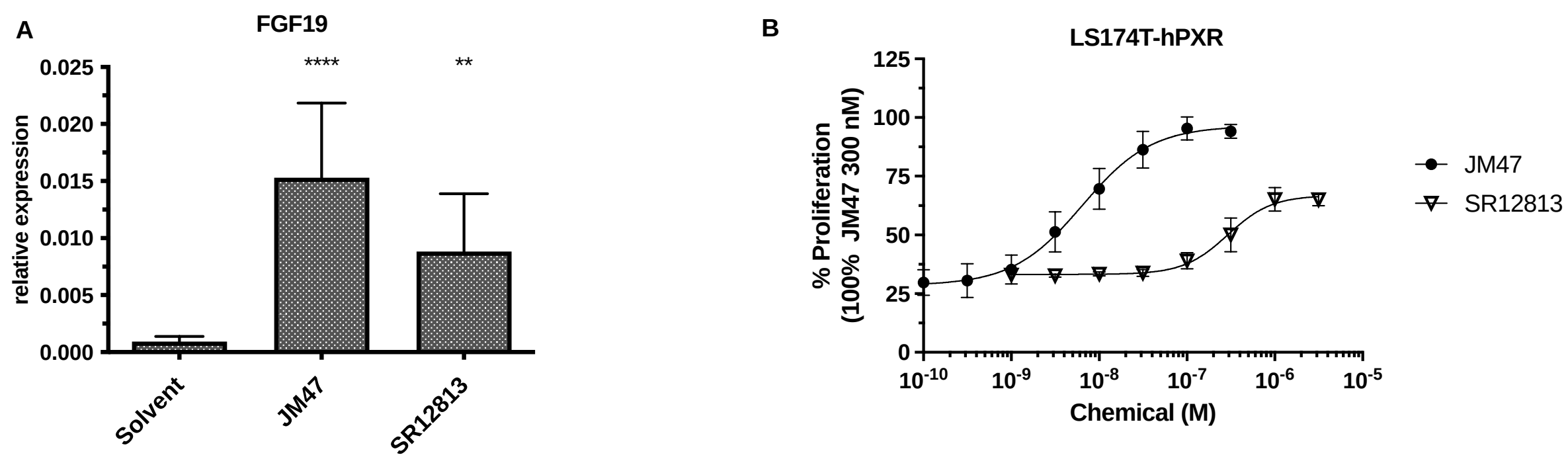

Supplemental Fig. 3. Effect of JM47 and SR12813 on FGF19 mRNA expression and proliferation in LS174T-PXR cells. (**A**) RT-qPCR of FGF19 mRNA expression was performed in LS174T-PXR cells treated with 0.1% DMSO (negative control), SR12813 3 μM and JM47 300 nM for 30 hours. After 30 h, RT-qPCR for FGF19 was performed and normalized to 28S levels. Results were obtained from four independent experiments performed in duplicate. Data are expressed as mean ± SD and compared with control cells treated with DMSO (0,1%). Statistical analysis was performed using a two-way ANOVA followed by Dunnett's post-hoc test for multiple comparisons, comparing each treatment condition to the DMSO control. p-values indicate comparisons of indicated groups versus the untreated group (*p ≤ 0.05, **p ≤ 0.01, ***p ≤ 0.001, ****p ≤ 0.0001). (**B**) Dose-response curves of LS174T-PXR cells proliferation induced by JM47 and SR12813. Cells were exposed to the treatments for 7 days in presence of SPA70 100 nM. Results are expressed as the percentage of the maximum proliferation induced by 300 nM JM47. Dose–response curves were fitted using a four-parameter sigmoidal model in GraphPad Prism. The Hill slope was treated as a variable parameter, the upper and lower asymptotes were left unconstrained, and no weighting was applied during the fitting procedure.

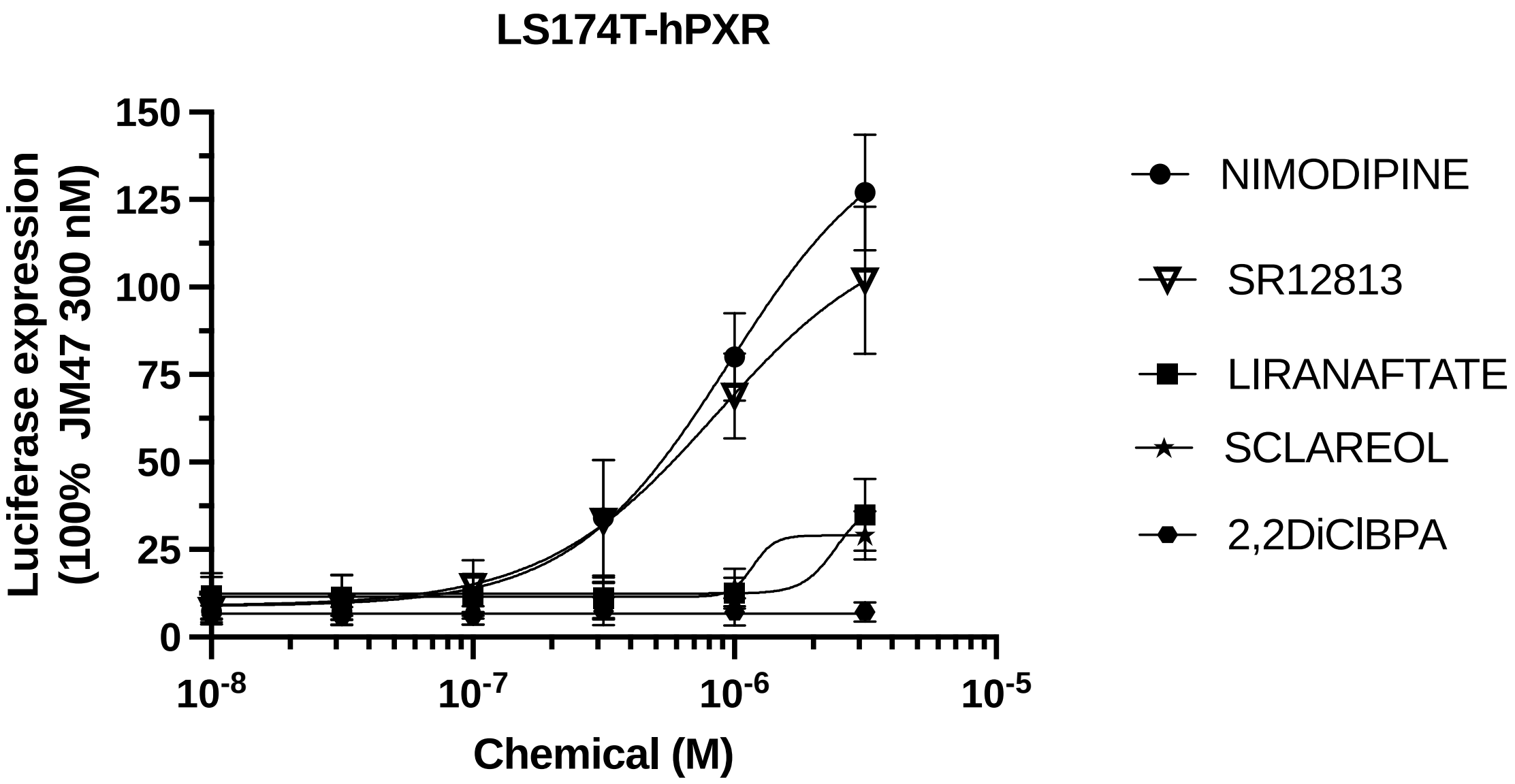

Supplemental Fig. 4. Luciferase expression in cell proliferation assays was measured in LS174T-PXR cells treated for 7 days with nimodipine, SR12813, liranaftate, sclareol, and 2,2′-diClBPA. To account for cell proliferation, relative light units (RLU) were normalized to absorbance values. Results are expressed as a percentage of the maximal luciferase activity induced by 300 nM JM47. Dose–response curves were fitted using a four-parameter sigmoidal model in GraphPad Prism. The Hill slope was treated as a variable parameter, the upper and lower asymptotes were left unconstrained, and no weighting was applied during the fitting procedure.

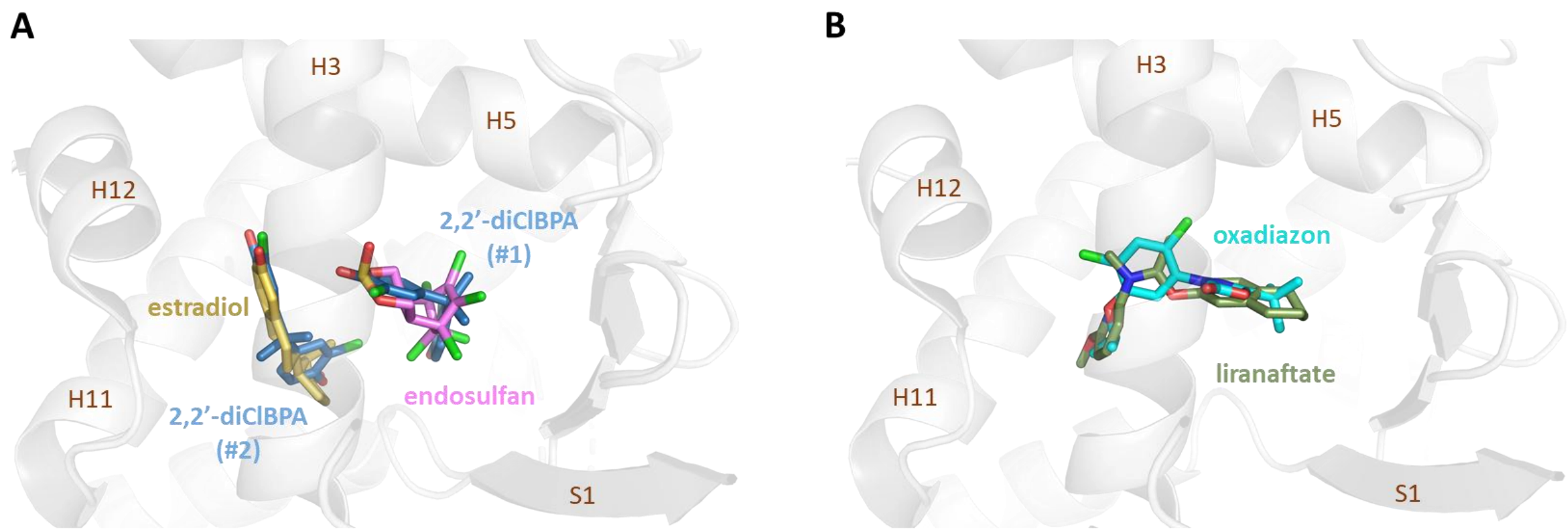

Supplemental Fig. 5. Binding modes of 2,2’-diClBPA and liranaftate to PXR. (**A**) Superposition of the PXR structure bound to 2,2’-diClBPA with the PXR structure bound to the pesticide endosulfan and the natural hormone estradiol (PDB code 7AXK). The binding site of 2,2’-diClBPA molecule #1 overlaps with that of endosulfan, whereas the binding site of 2,2’-diClBPA molecule #2 overlaps with that of estradiol. (**B**) Superposition of the PXR structures bound to liranaftate and oxadiazon (PDB code 7AXE), showing that the binding sites of the two compounds overlap.

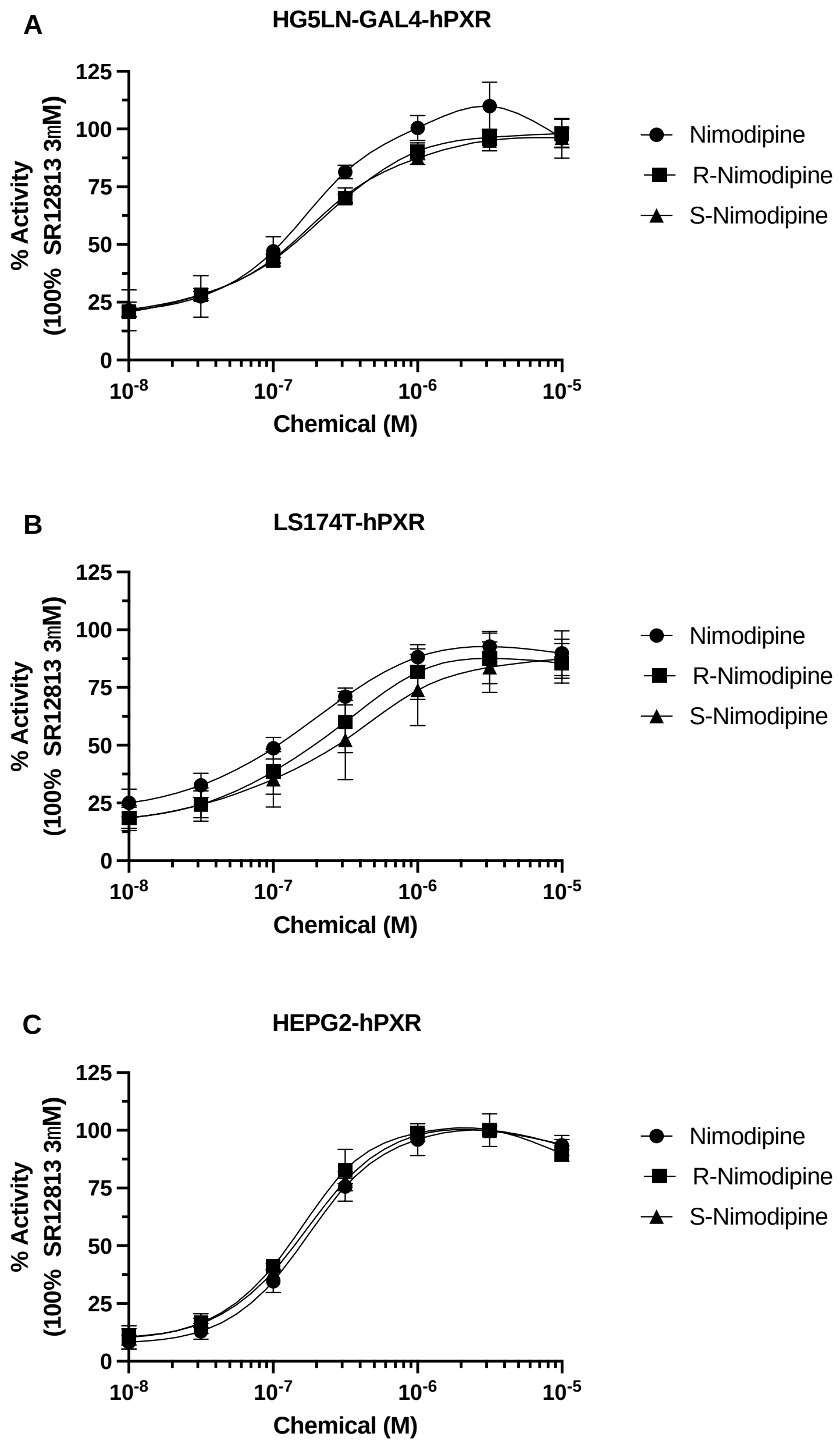

Supplemental Fig. 6. Transactivation assays of nimodipine enantiomers. Dose-response curves of luciferase expression induced by (R, S)-nimodipine, R-nimodipine, and S-nimodipine in HG5LN-GAL4-PXR (**A**), LS174T-PXR (**B**) and HepG2-PXR (**C**) cells. Cells were exposed to the treatments for 30 hours. Results are expressed as the percentage of the maximum luciferase activity induced by 3 µM SR12813. Transactivation dose-response curves were fitted using the sigmoid dose–response function of GraphPad Prism software. EC50 values were determined by nonlinear regression of the response versus log(concentration) data using a four-parameter sigmoidal model. The Hill slope was allowed to vary, the upper plateau was constrained to 100%, and no weighting was applied. EC50 values were first calculated for each biological replicate ($n \geq 3$). The geometric mean was then computed across all experiments, and 95% confidence intervals were subsequently determined.

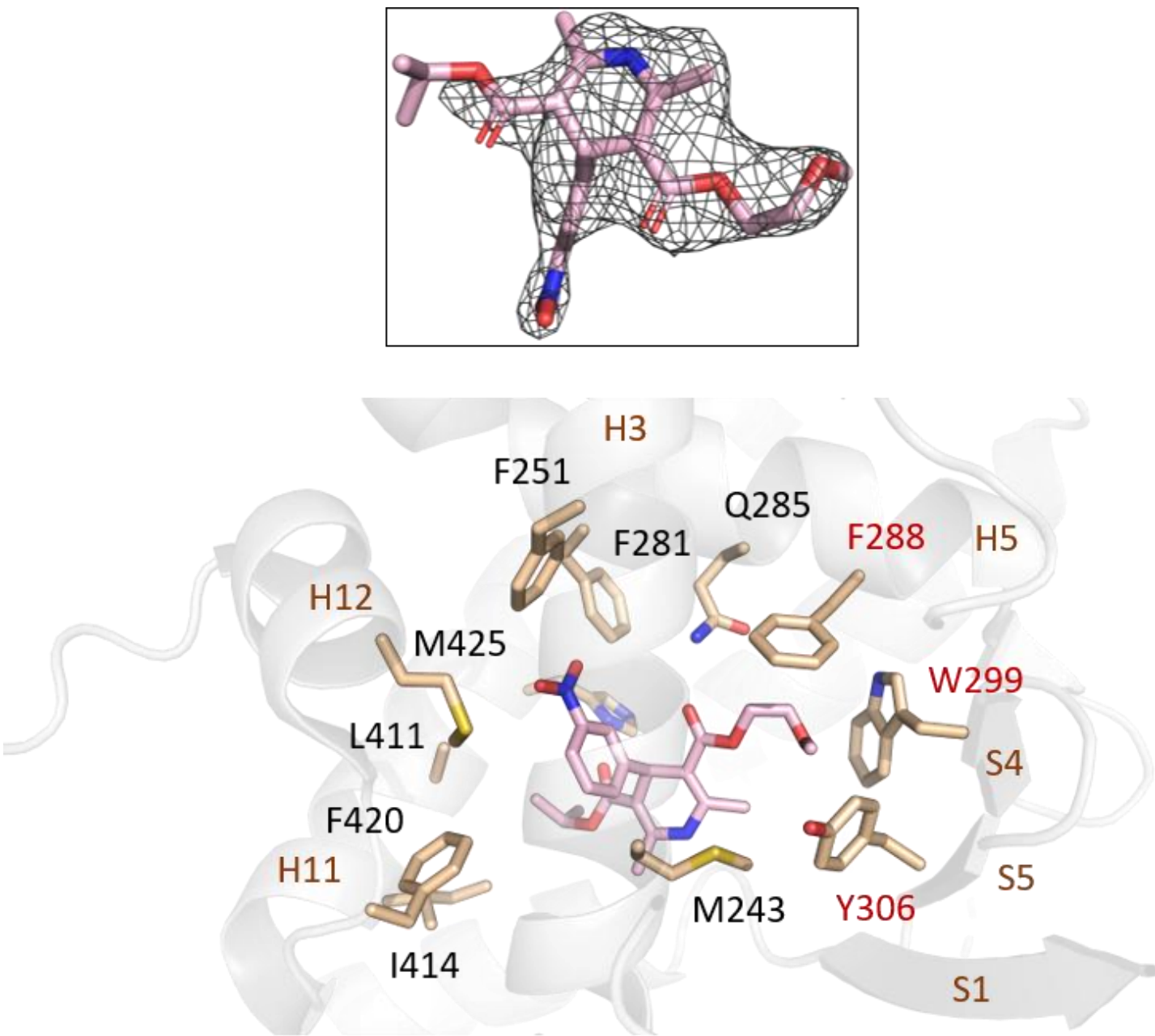

Supplemental Fig. 7. Close-up view of the ligand-binding pocket of PXR bound to S-nimodipine. Key PXR residues in contact with the ligand (cut-off 4.2 Å) are depicted. The inset shows the 2Fo-FC electron density map with S-nimodipine.

Supplemental Table 4. Number of experiments to determine transcriptional activities of chemicals in HG5LN-GAL4-PXR, LS174T-PXR and HepG2-PXR reporter cell lines.

**Compound**	**Number of experiments**		
	**HG5LN-GAL4-PXR**	**LS174T-PXR**	**HepG2-PXR**
SR12813 (Ref. agonist)	6	8	3
Liranaftate	3	3	3
Nimodipine	6	7	6
Sclareol	4	5	7
2,2'-diClBPA	3	3	3
SPA70 (Antagonist)	5	10	3

Supplemental Table 6. Number of experiments to determine proliferative activities of chemicals in LS174T-PXR reporter cell lines.

**Compound**	**Number of experiments**
SR12813 (Ref. agonist)	7
Liranaftate	4
Nimodipine	4
Sclareol	4
2,2'-diClBPA	3
SPA70 (Antagonist)	6

Supplemental Table 7. Primer sequences used in this study.

	**Forward (5' → 3')**	**Reverse (5' → 3')**
28S	CGATCCATCATCCGCAATG	AGCCAAGCTCAGCGCAAC
FGF19	AGATCAAGGCAGTCGCTCTG	AAAGCACAGTCTTCCTCCGA
CYP3A4	GCCTGGTGCTCCTCTATCTA	GGCTGTTGACCATCATAAAAG

Supplemental Table 8. Data collection and refinement statistics*

Ligand	2,2'-diClBPA	Liranaftate	Sclareol	Nimodipine
PDB ID	8CCT	8CH8	8CF9	9TWE
Wavelength (Å)	0.9677	0.6199	0.9762	0.6199
Resolution range (Å)	32.59 - 2.90 (3.00 - 2.90)	64.81 - 2.15 (2.23 - 2.15)	41.07 - 2.00 (2.07 - 2.00)	40.87 - 2.40 (2.49 - 2.40)
Space group	P 43 21 2	P 43 21 2	P 43 21 2	P 43 21 2
Unit cell (Å, °)	92.18 92.18 86.59 90 90 90	91.65 91.65 85.74 90 90 90	91.83 91.83 85.89 90 90 90	91.39 91.39 86.23 90 90 90
Total reflections	44,422 (4,531)	125,645 (4,316)	316,880 (30,780)	116,103 (11,666)
Unique reflections	8,519 (838)	18,337 (1,495)	25,385 (2,463)	14,745 (1,445)
Multiplicity	5.2 (5.4)	6.9 (2.9)	12.5 (12.5)	7.9 (8.1)
Completeness (%)	97.45 (99.76)	96.24 (76.40)	99.89 (99.96)	99.26 (99.93)
Mean I/sigma(I)	14.11 (2.42)	19.54 (1.00)	21.81 (3.79)	22.25 (2.74)
Wilson B-factor ($Å^2$)	71.41	52.53	37.62	60.85
R-merge	0.081 (0.604)	0.049 (0.569)	0.075 (0.650)	0.048 (0.585)
CC1/2	0.999 (0.803)	0.999 (0.786)	0.998 (0.899)	0.997 (0.859)
**Refinement**				
No. of reflections	8,495 (837)	19,560 (1,495)	25,374 (2,464)	14,725 (1,446)
R-work	0.205 (0.279)	0.206 (0.318)	0.210 (0.280)	0.225 (0.290)
R-free	0.259 (0.342)	0.228 (0.326)	0.247 (0.301)	0.269 (0.313)
No. of non-H atoms	2,147	2,272	2,314	2,188
macromolecules	2,098	2,181	2,160	2,085
ligands	38 (19 x 2)	23	22	30
solvent	11	68	126	43
Protein residues	267	275	271	267
RMS (bonds, Å)	0.006	0.003	0.002	0.003
RMS (angles, °)	1.14	0.72	0.53	0.60
Ramachandran (%)				
favored	96.93	98.14	98.48	98.47
allowed	2.68	1.86	1.52	1.53
outliers	0.38	0.00	0.00	0.00
B-factors ($Å^2$)	67.29	62.59	44.79	65.90
macromolecules	66.94	62.44	44.38	65.09
ligands	89.00	87.18	61.25	87.29
solvent	59.12	59.33	48.07	61.02

*Statistics for the highest-resolution shell are shown in parentheses.

Supplemental Table 9. EC50 and maximal activity values obtained in transactivation assays of dihydropyridine analogues. Values are the mean ± SD from three separate experiments.

	**HG5LN-GAL4-PXR**		**LS174T-PXR**		**HepG2-PXR**	
	Basal level cell line (%) 24±2		Basal level cell line (%) 27±3		Basal level cell line (%) 8±1	
Compound	EC50 (nM) (95% CI)	Max (%))	EC50 (nM) (95% CI)	Max (%)	EC50 (nM) (95% CI)	Max (%)
Cilnidipine	305 (267 to 348)	102±4	571 (310 to 1172)	78±2	778 (603 to 1010)	89±7
Felodipine	2409 (1502 to 5696)	47±3	1921 (1125 to 4747)	57±13	1594 (1283 to 2108)	53±1
Isradipine	1410 (949 to 2305)	72±4	659 (462 to 974)	78±2	523 (462 to 601)	87±3
Nicardipine	850 (621 to 966)	104±9	889 (755 to 1050)	84±3	714 (689 to 739)	95±4
Nilvadipine	607 (450 to 825)	91±5	599 (441 to 827)	83±3	360 (326 to 399)	93±10
Nimodipine	146 (96 to 217)	110±9	183 (100 to 301)	93±5	171 (140 to 207)	100±5
Nisoldipine	678 (371 to 1404)	75±4	981 (754 to 1389)	63±3	781 (481 to 1383)	70±7

Supplemental Table 10. EC50 (μM) and maximal activity values of transactivation assays of halogenated BPA analogues. Values are the mean ± SD from three separate experiments.

	**HG5LN-GAL4-PXR**		**LS174T-PXR**		**HepG2-PXR**	
	Basal level cell line (%) 24±2		Basal level cell line (%) 28±3		Basal level cell line (%) 8±1	
Compound	EC50 (μM) (95% CI)	Max (%)	EC50 (μM) (95% CI)	Max (%)	EC50 (μM) (95% CI)	Max (%)
BPA	18.2 (16.4 to 20.6)	50±5	21.2 (8.5 to 24.8)	46±7	45.5 (32.0 to 71.9)	24±2
2,2'-diClBPA	3.9 (2.5 to 6.4)	75±2	39.5 (30.8 to 53.8)	43±6	10.8 (10.1 to 11.6)	52±4
2-Br,2'-ClBPA	3.9 (3.8 to 4.2)	63±4	34.3 (12.6 to 47.7)	43±5	17.2 (15.8 to 18.9)	42±7
2,2'-diBrBPA	13.9 (12.4 to 16.2)	52±10	26.9 (22.5 to 33.5)	40±7	15.3 (14.9 to 15.8)	43±5
TClBPA	14.4 (12.6 to 17.0)	51±5	19.7 (17.8 to 22.2)	41±11	ND*	19±5
TBrBPA	13.4 (12.9 to 14.0)	51±4	23.1 (12.0 to 27.4)	36±2	ND*	16±1

*Not determined because of insufficient response for curve fitting.

Supplemental Table 11. Induction factors of CYP3A4 and FGF19 gene expression of nimodipine analogues. Values are the mean ± SD from three separate experiments.

Fold induction over DMSO (95 % CI)		
	CYP3A4	FGF19
SPA70	0.04 (0.01 to 0.16)	0.6 (0.3 to 1.3)
SR12813	20.9 (11.6 to 32.6)	10.7 (7.8 to 14.8)
Nimodipine	20.3 (9.1 to 35.5)	24.5 (12.1 to 39.5)
Liranaftate	29.7 (19.1 to 34.7)	17.1 (5 to 28.1)
Sclareol	30.4 (14.8 to 52.6)	19.4 (8 to 27.3)
2,2'-diClBPA	1.6 (0.7 to 3.4)	1.2 (0.6 to 2.2)
JM47	-	20.1 (16.2 to 25)

Supplemental Table 12. EC50 (μM) and maximal activity values of transactivation assays of nimodipine analogues. Values are the mean ± SD from three separate experiments.

	**HG5LN-GAL4-PXR**		**LS174T-PXR**		**HepG2-PXR**	
	Basal level cell line (%) 24±2		Basal level cell line (%) 28±3		Basal level cell line (%) 8±1	
Compound	EC50 (nM) (95% CI)	Max (%)	EC50 (nM) (95% CI)	Max (%)	EC50 (nM) (95% CI)	Max (%)
Nimodipine	146 (96 to 217)	110±9	183 (100 to 301)	93±5	171 (140 to 207)	100±5
R-Nimodipine	273 (212 to 348)	101 ± 1	159 (153 to 165)	92 ± 4	152 (124 to 185)	100 ± 2
S-Nimodipine	196 (177 to 217	99 ± 1	202 (132 to 294)	91 ± 2	155 (141 to 190)	100 ± 1

Supplemental information.

Plasmid maps used in this study.

XREM 3A4 LUC+ HYGRO

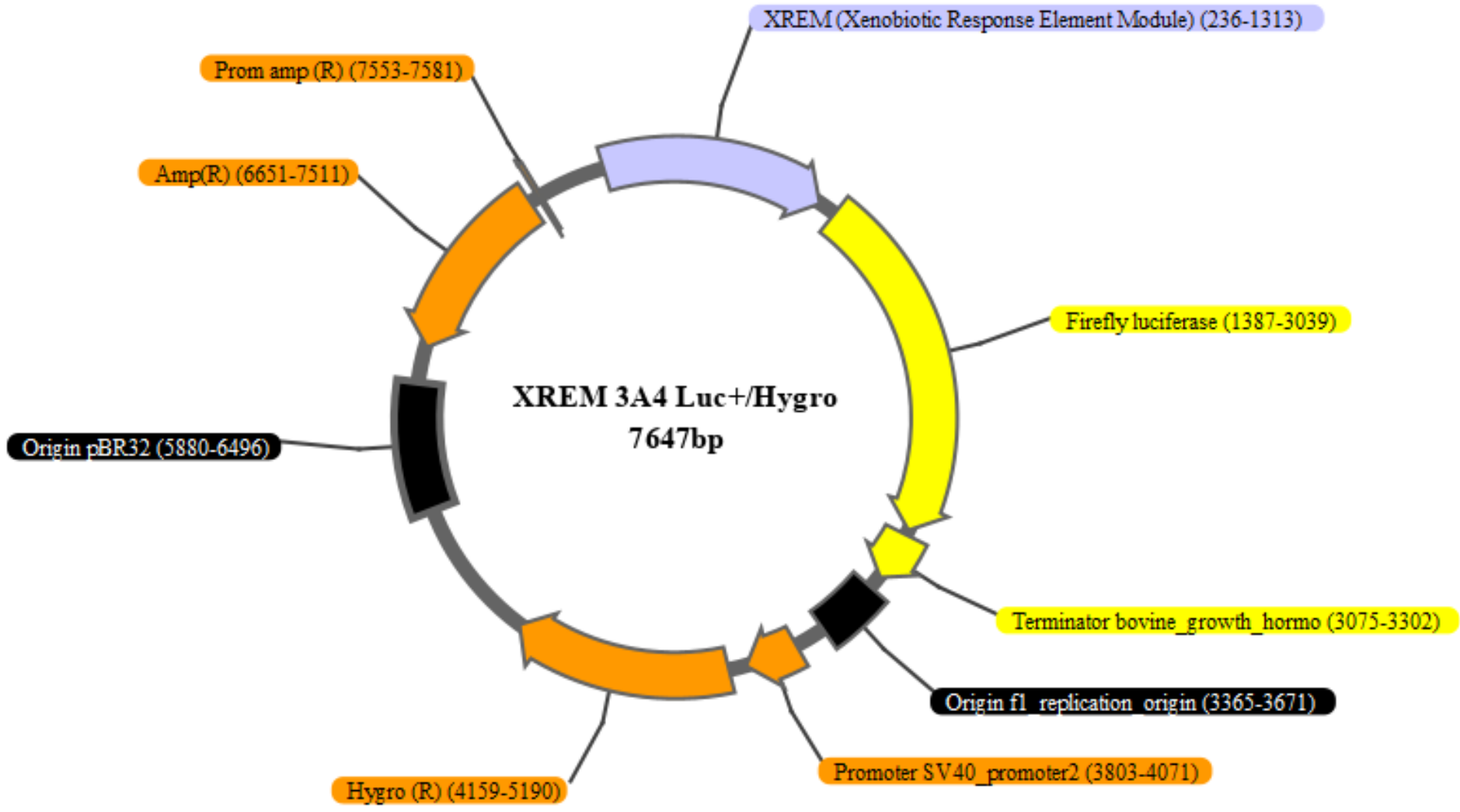

GAL4REx5 BGLOB LUC SV NEO

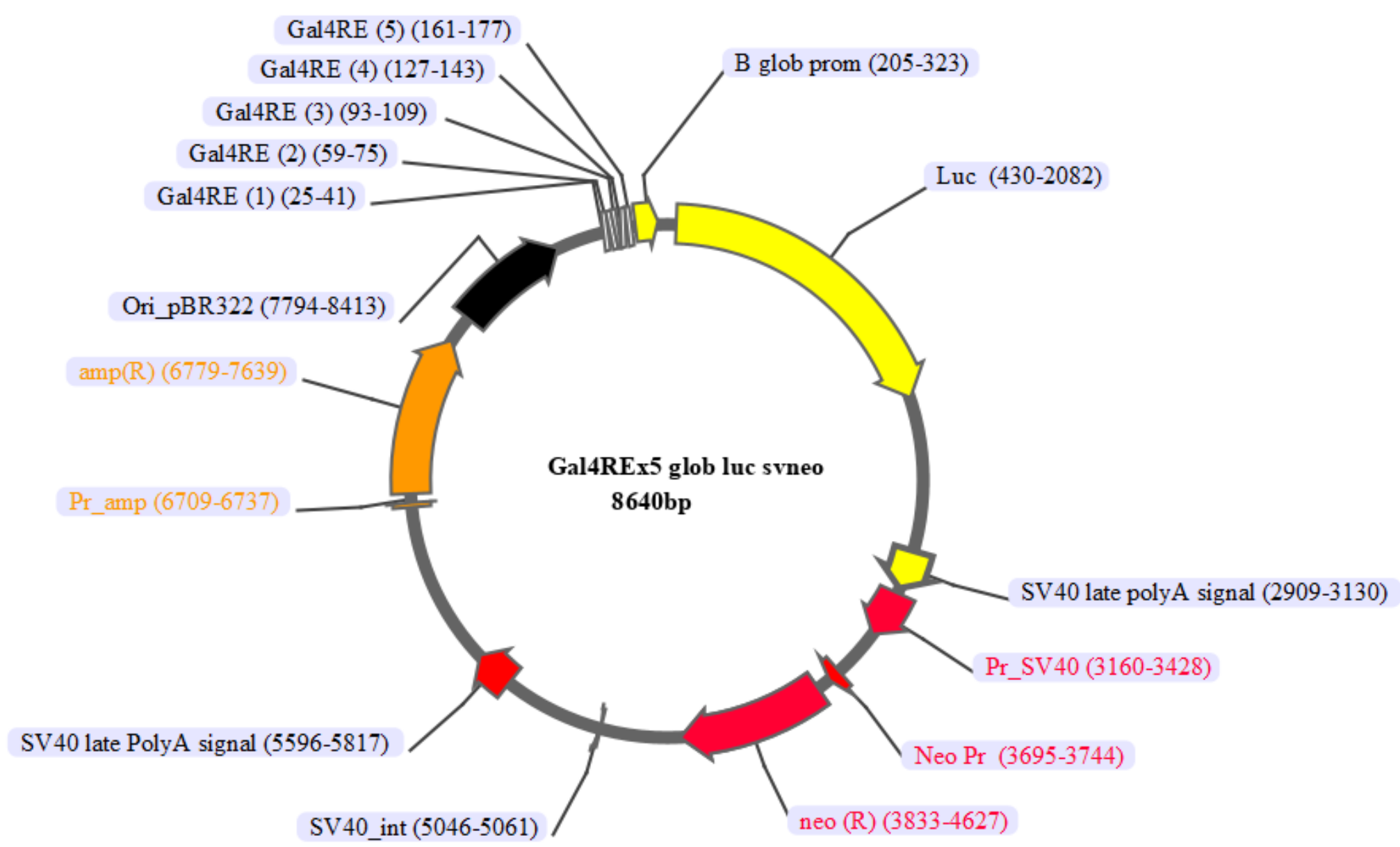

pcDNA3.1(+) hPXR NEO

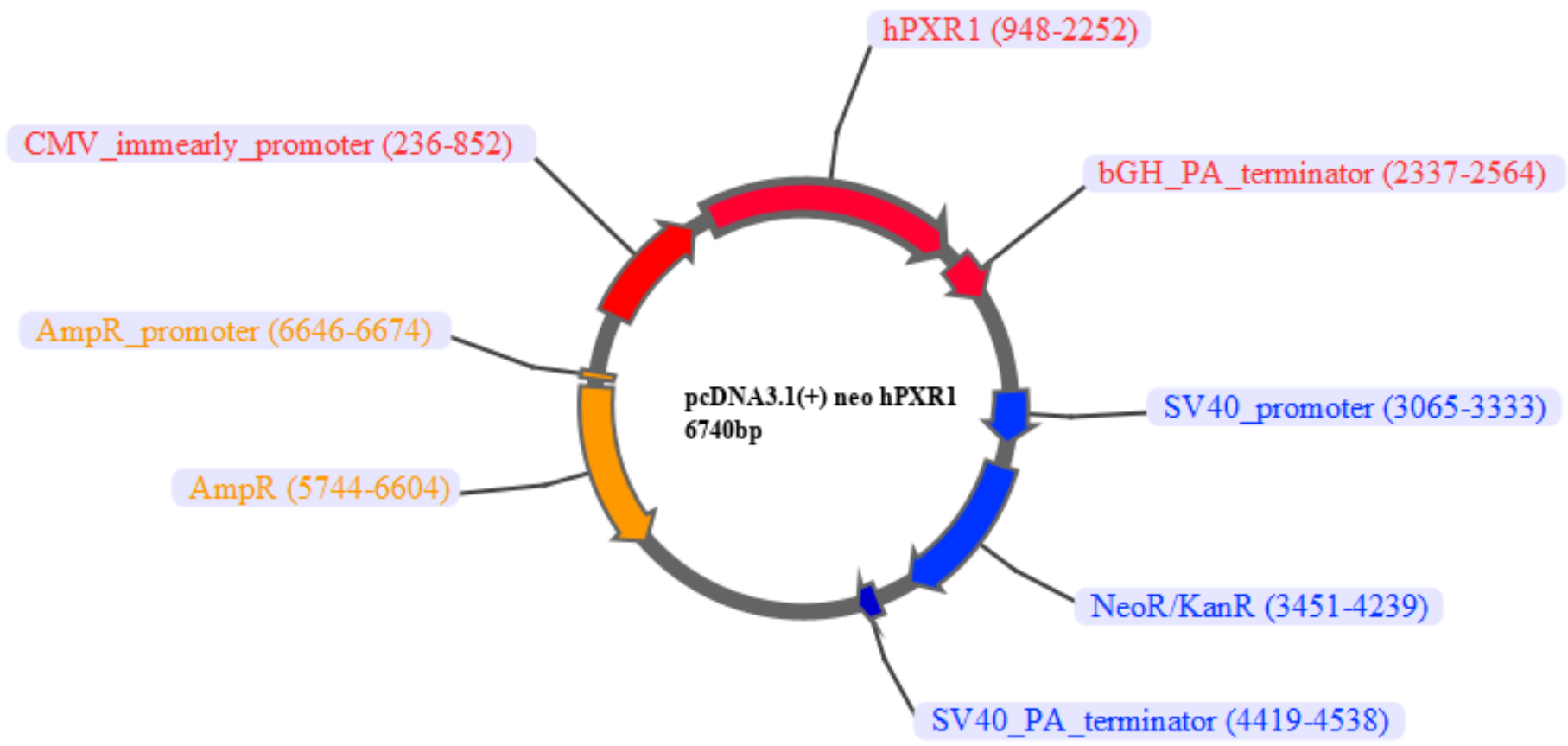

pSG5 GAL4(DBD) – PXR(LBD) PURO

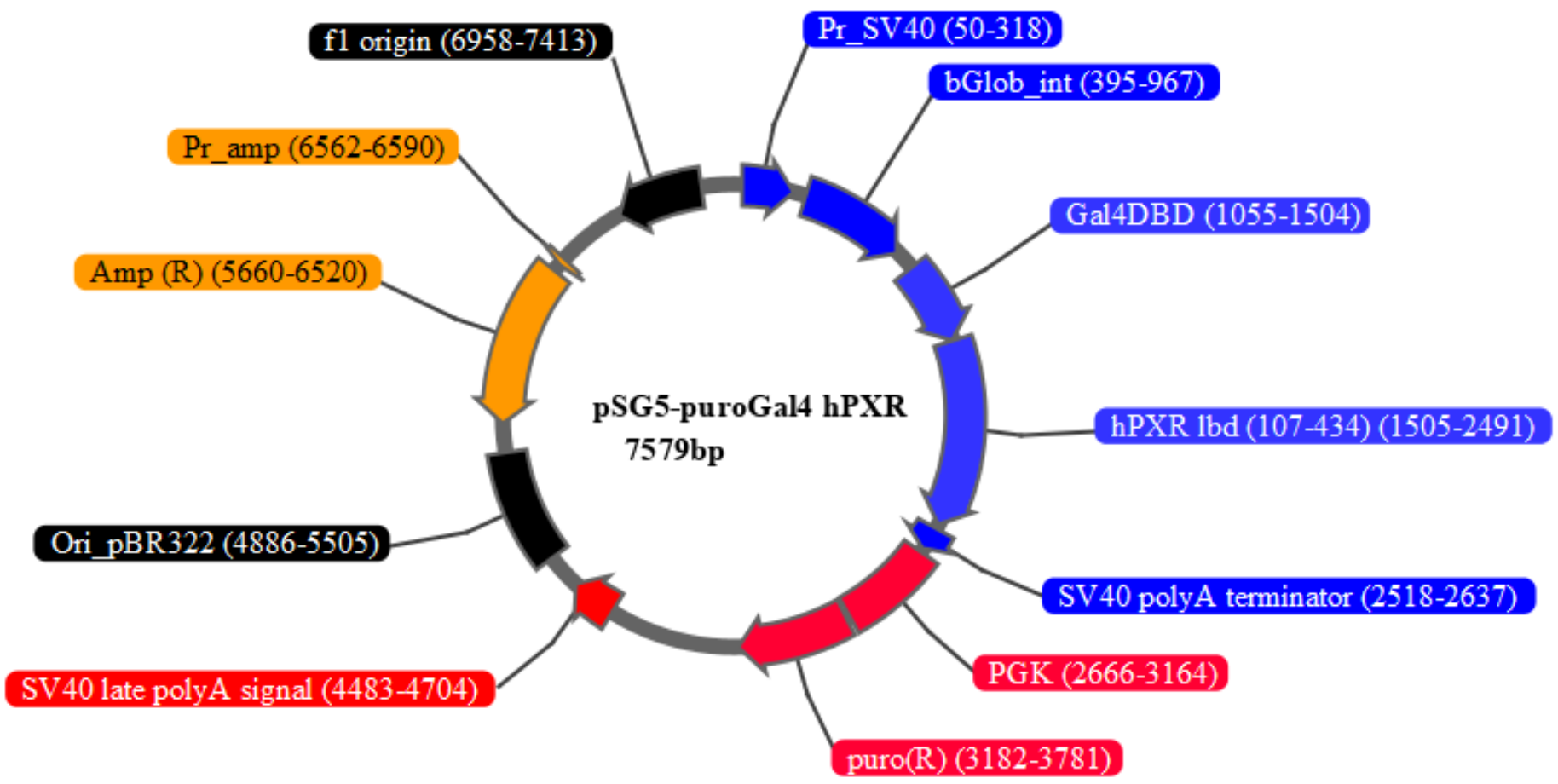

Supplement: Supplementary file 1 [file mmc3.pdf]
